# Bioprospection of the bacterial β-myrcene-biotransforming trait in the rhizosphere

**DOI:** 10.1007/s00253-023-12650-w

**Published:** 2023-07-05

**Authors:** Pedro Soares-Castro, Filipa Soares, Francisca Reis, Teresa Lino-Neto, Pedro M. Santos

**Affiliations:** 1 CBMA – Centre of Molecular and Environmental Biology, University of Minho, Campus de Gualtar, Braga, Portugal; 2 Present Address: Faculdade de Medicina, Instituto de Medicina Molecular, Universidade de Lisboa, Av. Prof. Egas Moniz, Lisbon, Portugal

**Keywords:** Cork oak rhizosphere, Eucalyptus rhizosphere, Monoterpene biotransformation, β-Myrcene, *Pseudomonas*, Integrative conjugative element

## Abstract

**Abstract:**

The biocatalysis of β-myrcene into value-added compounds, with enhanced organoleptic/therapeutic properties, may be performed by resorting to specialized enzymatic machinery of β-myrcene-biotransforming bacteria. Few β-myrcene-biotransforming bacteria have been studied, limiting the diversity of genetic modules/catabolic pathways available for biotechnological research. In our model *Pseudomonas* sp. strain M1, the β-myrcene catabolic core-code was identified in a 28-kb genomic island (GI). The lack of close homologs of this β-myrcene-associated genetic code prompted a bioprospection of cork oak and eucalyptus rhizospheres, from 4 geographic locations in Portugal, to evaluate the environmental diversity and dissemination of the β-myrcene-biotransforming genetic trait (Myr^+^).

Soil microbiomes were enriched in β-myrcene-supplemented cultures, from which β-myrcene-biotransforming bacteria were isolated, belonging to *Alphaproteobacteria*, *Betaproteobacteria*, *Gammaproteobacteria*, and *Sphingobacteriia* classes. From a panel of representative Myr^+^ isolates that included 7 bacterial genera, the production of β-myrcene derivatives previously reported in strain M1 was detected in *Pseudomonas* spp., *Cupriavidus* sp., *Sphingobacterium* sp., and *Variovorax* sp. A comparative genomics analysis against the genome of strain M1 found the M1-GI code in 11 new *Pseudomonas* genomes. Full nucleotide conservation of the β-myrcene core-code was observed throughout a 76-kb *locus* in strain M1 and all 11 *Pseudomonas* spp., resembling the structure of an integrative and conjugative element (ICE), despite being isolated from different niches.

Furthermore, the characterization of isolates not harboring the Myr^+^-related 76-kb *locus* suggested that they may biotransform β-myrcene via alternative catabolic loci, being thereby a novel source of enzymes and biomolecule catalogue for biotechnological exploitation.

**Key points:**

• *The isolation of 150 Myr*^+^ *bacteria hints the ubiquity of such trait in the rhizosphere.*

• *The Myr*^+^ *trait is spread across different bacterial taxonomic classes.*

• *The core-code for the Myr*^+^ *trait was detected in a novel ICE, only found in Pseudomonas spp.*

**Supplementary Information:**

The online version contains supplementary material available at 10.1007/s00253-023-12650-w.

## Introduction

Plant monoterpenes and their derivatives have been widely used as starting materials for the production of fine chemicals, because of their biotechnologically relevant organoleptic and therapeutic properties (Edris 2007; Bicas et al. 2009). The industrial exploitation of monoterpenes usually involves their chemical oxidation/hydroxylation and incorporation into aqueous formulations. Such strategy may be troublesome due to the high hydrophobicity and structural instability of monoterpenes, which often requires the utilization of surfactants and the presence of a second phase of organic solvents (Krings and Berger 1998; Schwab et al. 2013). These catalytic constrains may be overcome by resorting to the specialized enzymatic machinery of monoterpene-catabolizing microbes. They are able to quickly adapt to the hydrophobic character of monoterpenes, overcome the limited substrate availability, and modify the monoterpene backbone with high regio- and stereo-selectivity (Krings and Berger 1998). Nowadays, increasing research effort is being performed towards a more profitable bio-based transformation and catalysis, by prospecting ecological niches as source of novel cell factories and enzymes (Soares-Castro et al. 2021, and references therein).

The acyclic β-myrcene (7-methyl-3-methylene-1,6-octadiene) is one of the most promising monoterpenes, being used as starting material for the chemical synthesis of top selling flavors and fragrances (e.g., linalool, geraniol, citral, menthol), compounds with pharmacological potential (e.g., antimutagenics, analgesics, tyrosinase inhibitors) and also in the production of polymers, biodegradable surfactants, pheromones, and agrochemicals (Kauderer et al. 1991; Matsuura et al. 2006; Behr and Johnen 2009). Despite efforts to describe β-myrcene biotransformation in different models (*Pseudomonas putida* S4-2, *Pseudomonas* sp. strain M1, *Pseudomonas aeruginosa* PTCC 1074, *Rhodococcus erythropolis* MLT1, and *Castellaniella defragrans* 65Phen strains) (Narushima et al. 1981; Iurescia et al. 1999; Thompson et al. 2010; Esmaeili and Hashemi 2011; Lüddeke and Harder 2011; Soares-Castro et al. 2017), the majority of the studies lacked a holistic characterization of the β-myrcene catabolic pathways towards proper biotechnological exploitation. The knowledge regarding the repertoire of enzymes available in the environment for β-myrcene biotransformation is still scarce.

*Pseudomonas* sp. strain M1, isolated from soil sediments of the Rhine River (Netherlands), is able to use β-myrcene as sole carbon (myr^+^ trait) (Iurescia et al. 1999; Santos and Sá-Correia 2009; Soares-Castro and Santos 2015; Soares-Castro et al. 2017). The molecular basis of such trait was characterized through the combination of genome-wide, bioanalytical chemistry, molecular biology, and microbial physiology approaches which led to the description of a novel 28-kb genomic island (GI) associated with β-myrcene catabolism (Santos and Sá-Correia 2009; Soares-Castro and Santos 2015).

Moreover, a detailed phylogenetic analysis of the 28-kb GI showed (i) low homology levels to sequences available in public databases and (ii) the majority of genes comprised in the island shared significant identity with sequences from organisms that belong to a taxonomic class not related to *Pseudomonas* spp. In other words, the analysis suggested the 28-kb GI might have resulted from a modular-like assembly of DNA sequences acquired from different environmental sources, which were functionalized towards β-myrcene utilization in strain M1 (Soares-Castro and Santos 2015). These hints also suggested the existence of broader spectrum of bacteria able to metabolize/biotransform β-myrcene, when compared with the current knowledge. Such functional modules might hold biotechnological potential as targets for engineering strategies, by mimicking the transformations intended on the chemical catalysis of β-myrcene. Soares-Castro et al. (2017) have detected the production of myrcen-8-ol ((2E)-2-methyl-6-methylideneocta-2,7-dien-1-ol) and 4-methylhexanoic acid during the biotransformation of β-myrcene by M1 cells, which have been reported as insect pheromones and can be exploited as biocontrol agents, as well as the aroma compound 4-methyl-3-hexenoic acid (4-methylhex-3-enoic acid).

In nature, monoterpenes are mainly present in aerial organs of plants but have also been detected in the soil, derived from root exudates and accumulation of dead plant material (Wilt et al. 1993; Smolander et al. 2006; Lin et al. 2007). Previous studies report that accumulated soil monoterpenes might become available as carbon sources and modulate the dynamics of the soil microbiome and nutrient recycling (Misra and Pavlostathis 1997; Vokou et al. 2002; Smolander et al. 2006). Having this in mind, the soil can be considered a rich reservoir of novel gene products and metabolic pathways and a potential source of monoterpene biocatalysts for biotechnological research (Lee and Lee 2013).

The catalytic potential of soil microbial communities may be exploited by promoting the growth and enrichment of the microbial community of a soil sample towards the mineralization of a desired substrate (Steele and Stowers 1991). Despite the limitations of such strategies in underestimating the soil microbial diversity, due to singular growth requirements of the uncultured fraction, the cultivation-dependent approaches allow the physical isolation of strains presenting the desired metabolic trait, whose genetic traits can be further characterized.

Having in mind the limited knowledge regarding β-myrcene-biotransforming bacteria and the underlying genetic modules, in the present study, the bacterial communities of plant-associated soil samples, from 4 different geographic locations in Portugal, were enriched towards the biotransformation of β-myrcene. We aimed at the isolation of novel β-myrcene-catabolizing bacteria to broaden the current knowledge about the taxonomic context underlying the prokaryotic β-myrcene biotransformation, as well as to provide new insights regarding the environmental dissemination of the Myr^+^ trait, for future biotechnological exploitation.

## Materials and methods

### Soil collection and sample preparation

The soil sediments used in this study were collected in four different geographic locations in Portugal. Reis et al. (2019) provided soil samples derived from the rhizosphere of cork oak trees (*Quercus suber* L.) in Cabril (41°45′43.05″N 8°1′39.09″W) and Ermida (41°42′39.76″N 8°6′14.87″W), in Peneda-Gerês National Park (located in northern Portugal), as well as soil samples from the rhizosphere of cork oak trees in Grândola (38°11′32.37″N 8°37′11.41″W, located in southern Portugal). In Cabril, Ermida, and Grândola, five independent trees without signs of disease or infestation, separated at least 30 m from each other, were selected for soil sampling, under the middle of the cork oak canopy, resulting in 5 replicates per location (Reis et al. 2019). Soil samples were also collected in Guimarães (41°27′46.24″N 8°16′2.92″W, northern Portugal), from the rhizosphere of three eucalyptus trees (*Eucalyptus globulus* Labill.) separated by 10 m from each other (5 replicates). 

### Enrichment cultures of soil samples towards β-myrcene utilization

To perform the enrichment cultures, 10 g of soil replicates from each location were resuspended in 30 mL of 0.9% (w/v) NaCl, vortexed vigorously at 2500 × *g* for 10 min and the particles cleared by centrifuging at 1000 × *g* for 3 min. The cleared supernatants were centrifuged at 16,000 × *g* for 5 min to sediment microbial cells. The cells were resuspended in 1 mL of 0.9% (w/v) NaCl, and the replicates of each location were pooled into a single aliquot to inoculate the enrichment cultures (1 per each location). The microbial growth of soil suspensions was promoted during 48 h, in 50 mL of Mineral Medium (MM) (Soares-Castro and Santos 2015) in 250 mL flasks, supplemented with 0.05% (w/v) yeast extract, 40 mM glucose, and 40 mM pyruvate, at 30 °C and 200 rpm, to broaden the range of culturable bacteria retrieved from the soil suspensions (e.g., copiotrophs). Afterwards, 5 mL of the enrichment cultures were transferred to 50 mL of MM supplemented with 0.05% (w/v) yeast extract and with the saturating volume of 100 μL of β-myrcene [Acros Organics; CAS 123–35-3; log Kow: 4.17; water solubility at 25 °C of 5.6 mg L^−1^ or 41.1 μmol L^−1^ (Soares-Castro and Santos 2015); a volume of 100 μL corresponds to 130 mg L^−1^, 47.8 μmol in solution (Soares-Castro et al. 2017)], at 30 °C and 200 rpm, envisaging the enrichment and isolation of β-myrcene-biotransforming bacteria.

The amount of β-myrcene used in the experiments assured constant water phase saturation with the monoterpene to avoid carbon starvation effects. The flasks used with β-myrcene-supplemented cultures were sealed with rubber stoppers. During the enrichment approach, the optical density measured at 600 nm (OD_600 nm_) and pH values were monitored every 3 days, followed by supplementation of β-myrcene and correction of pH to values ranging from 6.5 to 7.0.

### Isolation and identification of β-myrcene-biotransforming bacteria

Putative β-myrcene-biotransforming bacteria were isolated between 6 and 30 days of enrichment. Bacteria cells were plated on MM agar supplemented with 0.05% (w/v) yeast extract and saturating amount of β-myrcene placed on the plate lid. A set of colonies representing different types of morphologies were isolated, and their ability to use β-myrcene as carbon source (the Myr^+^ trait) was evaluated in culture tubes at 30 °C and 200 rpm, containing 10 mL liquid cultures of MM with 0.05% (w/v) yeast extract, supplemented with the saturating 50 μL of β-myrcene (65 mg L^−1^, 23.9 μmol in solution), and prepared with an initial OD_600 nm_ value of 0.1. Cultures of MM + 0.05% (w/v) yeast extract, without supplementation with the monoterpene, were used as controls, to differentiate the β-myrcene-supported growth from residual growth supported by other constituents of the culture medium.

The taxonomic identification of the β-myrcene-biotransforming soil isolates was performed by Sanger sequencing of the 16S rRNA gene. Genomic DNA was extracted using the Wizard Genomic DNA Purification Kit (Promega), and the 16S rRNA gene was amplified by PCR, using the conserved Bacteria primer 8F (5′AGAGTTTGATCCTGGCTCAG 3′) (Gardener et al. 2000). Amplification was performed according to the following protocol: initial denaturation step at 96 °C for 3 min, 30 cycles of denaturation at 96 °C for 30 s, annealing at 50 °C for 30 s and extension at 72 °C for 1 min and 30 s, followed by a final extension step at 72 °C for 5 min. The PCR fragments were sequenced with an automatic ABI GeneAmp PCR System 9700 (GENEWIZ Ltd.) and the trimmed DNA sequences comprising the variable regions V2 to V4 (approximately 615 bp), used for taxonomic identification with the RDP server (Cole et al. 2013). The 16S rRNA gene sequences obtained from Sanger sequencing were aligned with MAFFT version 7 (Katoh and Standley 2013), and the maximum likelihood phylogenetic midpoint rooted tree was generated by using PhyML version 3.0 (Guindon et al. 2010), with 1000 bootstrap sets, the GTR (General Time Reversible) as the best-fit model of nucleotide substitution (evaluated by the W-IQ-Tree tool; Trifinopoulos et al. 2016), kappa estimated, 4 substitution rate categories, gamma distribution parameter estimated, BIONJ starting tree, with optimization of topology, branch lengths and rate parameter.

In this work, isolates obtained from Cabril, Ermida, Grândola, and Guimarães soils are designated as “UMC,” “UME,” “UMG,” and “UMA,” respectively.

### Growth kinetics of selected Myr^+^ isolates

The ability to use β-myrcene as carbon source, by Myr^+^ isolates belonging to different bacterial genera and showing different phenotypic traits, was validated in 50 mL cultures of MM, supplemented with 0.05% (w/v) yeast extract and the saturating amount of 100 μL of β-myrcene as carbon source, in 250 mL flasks sealed with rubber stoppers, at 30 °C and 200 rpm.

The cultures were prepared to an initial OD_600 nm_ value of 0.1. Similarly, control cultures of MM + 0.05% (w/v) yeast extract, without supplementation with β-myrcene, were used to distinguish the β-myrcene-supported growth (threshold of OD_600 nm_ at 8 h and 24 h > 0.2) from residual growth. *Pseudomonas* sp. strain M1 was used as reference to evaluate and compare growth kinetic parameters of the isolates. The maximum specific growth rate of each isolate using β-myrcene as carbon source (μ_myr_) was estimated using the standard equation μ_myr_ = ln(N2/N1)/t2-t1, in which “N1” and “N2” correspond to the variation of the cell abundance measured as OD_600 nm_, at two different time-points “t1” and “t2,” during the initial hours of the exponential phase. The pH values were monitored after 8 h and 24 h of growth to assess alterations of the chemical properties of the growth medium, as previously described in M1-grown cultures supplemented with β-myrcene as sole carbon source (Santos and Sá-Correia 2009; Soares-Castro et al. 2017).

The hierarchical clustering with growth parameters of each isolate (μ_myr_, OD_600 nm_ registered at approximately 8 h and 24 h of growth, pH values registered at approximately 24 h of growth) was performed based on the calculated Canberra distances and UPGMA agglomeration method, with the *hclust* function from the core-package “stats,” version 4.0.3, of the R statistical software (R Core Team 2013).

### Monitoring the activation of the promoter P5 of the β-myrcene hydroxylase MyrH of *Pseudomonas* sp. strain M1 in the Myr^+^ soil isolates

The GFP-based promoter-probe pSEVA637-P5 (Gm^R^; pBBR1 broad-host origin of replication) was mobilized to selected Myr^+^ soil isolates to evaluate their ability to activate the promoter P5 of the gene coding for the β-myrcene hydroxylase MyrH of *Pseudomonas* sp. strain M1 (*MRY70_06145*) (Soares-Castro et al. 2017).

The isolates harboring the promoter-probe were cultured in 10 mL MM supplemented with 0.05% (w/v) yeast extract, 40 mM glucose, 40 mM pyruvate, and 30 μg mL^−1^ of gentamicin. The reporter system was induced when cells reached an OD_600 nm_ of 0.3, by supplementing the culture medium with a saturating amount of 50 μL of β-myrcene (65 mg L^−1^, 23.9 μmol in solution). The β-myrcene-dependent activity of the promoter P5 was quantified at 24 h after β-myrcene supplementation, by fluorometric detection in a Qubit 3.0 fluorometer and expressed as the fold induction of GFP fluorescence normalized by the culture OD_600 nm_ between β-myrcene-induced and non-induced cells.

### Qualitative GC–MS analysis of β-myrcene-derivatives from cultures of representative Myr^+^ isolates

A panel of selected Myr^+^ isolates was cultured in 50 mL of MM with 0.05% (w/v) yeast extract, supplemented with the saturating amount of 100 μL of β-myrcene as sole carbon source. The cultures were prepared to an initial OD_600 nm_ value of 0.1 and were collected in the mid-exponential growth phase (OD_600 nm_ ranging from 0.4 to 0.5), for the following identification of the respective β-myrcene biotransformation products in the culture’s supernatant, according to Soares-Castro et al. (2017). Cultures of the wild-type strain of *Pseudomonas* sp. M1 were used as a reference.

A total of 50 mL of the supernatant (the whole culture) was collected, mixed with 10 mL ethyl acetate and vigorously shaken for 5 min. For each strain, 3 replicates of the extracts were produced for analysis. The ethyl acetate extracts were dried with nitrogen gas stream, dissolved in 1 mL of hexane and concentrated with a nitrogen gas stream to a final volume around 500 μL, for subsequent gas chromatography analysis coupled to mass spectrometry (Soares-Castro et al. 2017). Control metabolite profiles were prepared by using (i) pure β-myrcene standard in hexane; (ii) culture supernatants from cells grown with a mixture of 40 mM glucose and 40 mM pyruvate as sole carbon sources, without supplementation with terpenes; and (iii) abiotic control of MM with β-myrcene supplementation, without cells (Soares-Castro et al. 2017).

The gas chromatography analysis of the supernatants from biotransformation experiments was performed on a GC–MS Bruker Scion 436 with single quadrupole module, coupled to a CP8400 automated sampler and equipped with a Shimadzu RXi-5 Sil apolar capillary column (1,4-bis(dimethylsiloxy)phenylene dimethyl polysiloxane; 30 m × 0.25 mm I.D. × 0.25 μm film thickness). Helium was used as carrier gas at a flow rate of 1.0 mL min^−1^, and 1 uL of sample was injected in splitless mode. The column temperature was initially kept at 60 °C for 1 min and then gradually increased to 250 °C at a rate of 10 °C min^−1^, with a final hold time of 2 min (Soares-Castro et al. 2017). The mass spectra were acquired with an electron impact ionization voltage of 70 eV, scanned in the m/z range of 40–500 atomic mass unit. The baseline of the obtained chromatograms was defined with the statistics-sensitive non-linear iterative peak-clipping algorithm, which showed to be the best fit for the shape of different peak-free regions throughout the chromatogram (Soares-Castro et al. 2017).

### Metabolite identification

The total ion chromatograms were deconvoluted with the AMDIS tool version 2.71 (Stein 1999) to generate the ion spectra for every integer m/z component in the data acquisition range of atomic mass units. The deconvoluted m/z spectra whose intensity values increase, maximize, and decrease together are derived from the same chemical species, thus comprise its ion profile used for compound identification. The ion profile of each chemical species (here defined as the query peak) was obtained by considering the ion intensity at the query peak maximum and subtracting the intensity of the m/z components from the region of the GC–MS spectrum at the beginning and end of the query peak (to remove any influence derived from background ions of the matrix, and/or from adjacent peaks on either side of the query peak). Similarly, the ion profile of overlapped peaks, corresponding to co-eluted compounds, was manually filtered by subtracting the intensity of the m/z components of the contaminant peak from the ion profile of the query peak.

The identification of terpene-derivatives was performed based on (i) the NIST MS Search tool version 2.0 libraries (mainlib comprising 163,198 spectra and Wiley comprising 228,996 spectra) and (ii) by comparing calculated retention indices (RI) with those reported in the literature and/or from the National Institute of Standards and Technology (NIST) Mass Spec Data Center (Wallace 2023). Retention indices of each query compound were determined by sampling a mixture of n-alkanes (C10–C28) under the same GC–MS conditions (van Den Dool and Kratz 1963).

### Genome drafts of β-myrcene-biotransforming isolates

β-Myrcene-biotransforming soil isolates were selected for whole-genome sequencing, to determine their β-myrcene-associated genetic trait. The genomic DNA of the isolates was processed according to Illumina instructions to generate Nextera XT paired-end libraries. Isolates UMC13, UMC65, UMC76, UME9, UME65, UME77, and UME83 were sequenced using the Illumina MiSeq platform (2 × 250 bp), and isolates UMC631, UMC3103, UMC3106, UMC3129, UMA601, UMA603, and UMA643 were sequenced using the Illumina NextSeq500 platform (2 × 150 bp), at Instituto Gulbenkian de Ciência (IGC).

All raw paired-reads obtained from high throughput sequencing were initially corrected for sequencing errors with the k-spectrum-based BLUE tool set (Greenfield et al. 2014). Corrected read datasets were then filtered based on Phred quality scores (Ewing and Green 1998), and eventual adapter contamination, low quality, and ambiguous nucleotides were trimmed off by using the fastq-mcf tool (Aronesty 2011).

The derived filtered reads were then de novo assembled with the Unicycler pipeline (Wick et al. 2017), based on the SPAdes assembler, with default parameterization. Genome annotation was performed using the NCBI’s Prokaryotic Genomes Annotation Pipeline version 4.8 (Angiuoli et al. 2008).

### Comparative genomics between the 14 sequenced isolates and reference strains

The genome-based taxonomy was assigned to the sequenced isolates with the Bacsort toolset (https://github.com/rrwick/Bacsort), by estimating the average nucleotide identity (ANI) with FastANI version 1.33 (Jain et al. 2018), to determine the most suitable phylogenetically closer reference strains for the following species-level comparative genomics analyses. The following reference genomes were retrieved from NCBI using the Bacsort toolset and based on the ANI obtained: *Cupriavidus basilensis* strain DSM 11853 (GenBank accession GCF_008801925.2), *Pseudomonas protegens* strain CHA0 (GenBank accession NC_021237.1), *Sphingobacterium paramultivorum* strain BIGb0170 (GenBank accession CP058555) and *Variovorax ginsengisoli* strain S09.D (GenBank accession GCF_006438845.1).

### Assessing the conservation of the 28-kb β-myrcene core-code of strain M1 in 11 *Pseudomonas* isolates

The structural organization and nucleotide conservation of the genetic modules of M1-GI in the 11 isolates UMC65, UMC76, UMC631, UMC3103, UMC3106, UMC3129, UME65, UME83, UMA601, UMA603, and UMA643 was assessed by aligning the nucleotide sequence of the homologous *loci* in the CLC Genomics Workbench software (version 22.0.2, QIAGEN) with default parameters. The BLASTP-based homology search of the genes surrounding the 28-kb GI from M1 strain, putatively involved in DNA mobilization processes, was performed by using sequences described in the literature or retrieved from the ICEberg database (Bi et al. 2011).

## Results

### The isolation of bacterial strains harboring the Myr^+^ trait in the 4 Portuguese soils

In the present study, we prospected soil samples from the rhizosphere of cork oak trees and eucalyptus trees, of 4 different geographic locations in Portugal (UMC—Cabril; UME—Ermida; UMA—Guimarães; UMG – Grândola; Table S1), to isolate novel β-myrcene-catabolizing bacteria with biotechnological potential for β-myrcene biotransformation. The bacterial communities from the different soils were enriched during 30 days, in minimal medium supplemented with saturating amounts of β-myrcene as carbon-source, and the screenings for putative β-myrcene-biotransforming bacteria were carried out at different enrichment timepoints (between 6 and 30 days). The taxonomic identification was performed by Sanger sequencing of the 16S rRNA gene (Table S2). A total of 150 Myr^+^ Gram-negative bacteria were isolated and included members of the classes *Alphaproteobacteria* (2 *Agrobacterium* sp.), *Betaproteobacteria* (4 *Achromobacter* sp., 5 *Burkholderia* sp., 11 *Cupriavidus* sp., 2 *Delftia* sp., 2 *Variovorax* sp.), *Gammaproteobacteria* (1 *Acinetobacter* sp., 2 *Citrobacter* sp., 10 *Enterobacter* sp., 6 *Lelliottia* sp., 63 *Pseudomonas* sp., 3 *Raoultella* sp., 31 *Serratia* sp., 4 *Shigella* sp.), and *Sphingobacteriia* (4 *Sphingobacterium* sp.), as highlighted in Table 1 (the corresponding phylogenetic contextualization is shown in Fig. S1, Fig. S2 and Table S2).

**Table 1:** Overview of the bacterial taxa of the 150 soil isolates obtained from the enrichment cultures with β-myrcene selection. Their associated geographic location, taxonomic identification and phenotypic information (growth yield and pH at 24 h of culture in β-myrcene-supplemented medium) are listed in Table S2. Their phylogenetic clustering is shown in Fig. S1 and Fig. S2. Soil location: C, Cabril; E, Ermida; G, Grândola; A, Guimarães

Bacterial classes	Bacterial genera	Soil location	No. of isolates
*Alphaproteobacteria*	*Agrobacterium-Rhizobium group*	A	2
*Betaproteobacteria*	*Achromobacter* sp.	C, E, G	4
	*Burkholderia* sp.	C, G	5
	*Cupriavidus* sp.	C, E	11
	*Delftia* sp.	E	2
	*Variovorax* sp.	C	2
*Gammaproteobacteria*	*Acinetobacter* sp.	G	1
	*Citrobacter* sp.	G	2
	*Enterobacter* sp.	C, E, G, A	10
	*Lelliottia* sp.	G, A	6
	*Pseudomonas* sp.	C, E, G, A	63
	*Raoultella* sp.	G	3
	*Serratia* sp.	C, E, G, A	31
	*Shigella* sp.	G	4
*Sphingobacteriia*	*Sphingobacterium* sp.	C, E	4
**Total**			**150**

From the selected colonies, *Pseudomonas* spp. comprised 42.8% of all isolated bacteria, accounting for approximately 16%, 15%, 10%, and 4% of the retrieved isolates from Cabril, Ermida, Grândola, and Guimarães, respectively. The taxonomic classification of the 63 *Pseudomonas* spp*.* resulted in 3 distinct phylogenetic groups: 19 *P. putida*-like isolates, a second group of 12 *P. protegens-*like isolates, and a third group comprised by 26 isolates clustered with *Pseudomonas* sp. strain M1.

Members of the genera *Serratia* and *Enterobacter* were also isolated from all 4 locations, although with relative frequencies less than 10% and 3%, respectively, for each soil. On the contrary, the isolated *Variovorax* spp. were only retrieved from Cabril, and *Delftia* spp. were only retrieved from Ermida. Members of the genera *Acinetobacter*, *Citrobacter*, *Raoultella*, and *Shigella* were isolated from Grândola forests. *Agrobacterium* isolates were obtained from the eucalyptus rhizosphere of Guimarães.

### *Pseudomonas* isolates showed higher μ_myr_ in β-myrcene supplemented cultures

We validated the ability to use β-myrcene as carbon source in 43 Myr^+^ isolates, with different phenotypic traits (e.g., growth yield) and different genomic backgrounds, carried out in 50 mL batch cultures, to evaluate and compare their growth kinetics with the reference strain M1.

**Fig. 1:**
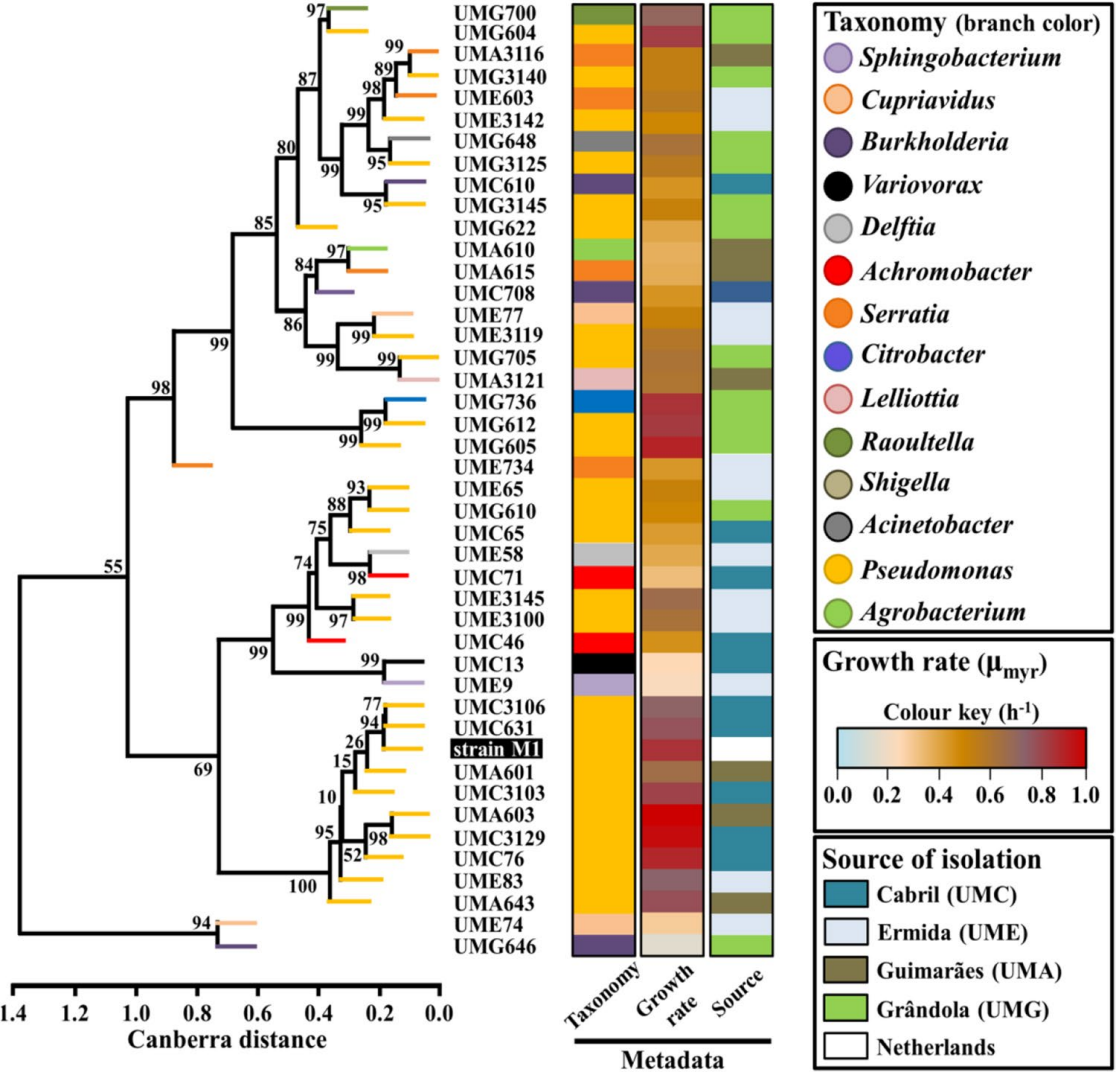
Clustering of 43 Myr^+^ isolates from the 4 geographic locations, based on the physiological characterization. The hierarchical clustering was performed with the calculated Canberra distances and UPGMA agglomeration, based on the estimated maximum specific growth rate (μ_myr_), OD_600 nm_ at 8 h and 24 h of growth, and pH at 24 h of growth. Isolate codes: “UMC,” Cabril; “UME,” Ermida; “UMG,” Grândola; “UMA,” Guimarães. Bootstrap values presented as percentage are shown in the tree branches. Colored columns summarize the metadata associated with each isolate: (i) taxonomy based on 16S rRNA gene Sanger sequencing, (ii) ranges of μ_myr_ in MM supplemented with 0.05% (w/v) yeast extract and β-myrcene, and (iii) source of isolation. Information about the 43 Myr^+^ isolates is detailed in Table S3

As shown in Fig. 1 and Table S3, the reference strain M1 registered a maximum specific growth rate (μ_myr_) of 0.85 ± 0.06 h^−1^ and OD_600 nm_ values > 1.0 in the culture conditions used, being only outperformed by the phylogenetically close *Pseudomonas* isolates UMA603 (0.98 ± 0.09 h^−1^), UMC3129 (0.95 ± 0.01 h^−1^), and UMC76 (0.88 ± 0.03 h^−1^).

The remaining 6 *Pseudomonas* isolates clustered with the strain M1 (clade G13 in Fig. S2) and reached similarly high OD_600 nm_ values after 8 h of growth, with μ_myr_ ranging from 0.65 ± 0.05 h^−1^ in isolate UMA601 to 0.81 ± 0.04 h^−1^ in isolate UMC3103. Based on the measured growth kinetics parameters (estimated μ_myr_, OD_600 nm_, and pH), the clustering profile obtained for strain M1 and the isolates UMA601, UMA603, UMA643, UMC76, UMC631, UMC3103, UMC3106, UMC3129, and UME83 (Fig. 1) was similar to the phylogenetic clustering from Fig. S2 (shared 100% nucleotide homology of the sequenced 16S rRNA gene fragment).

The *Pseudomonas* sp. isolate UMG605 and the *Citrobacter* sp. isolate UMG736 also registered maximum specific growth rates comparable to the μ_myr_ of strain M1 (0.89 ± 0.14 h^−1^ and 0.85 ± 0.03 h^−1^, respectively), however, associated with the production of less biomass. Whereas members of the *Pseudomonas* genus seemed to registered higher μ_myr_, values below 0.4 h^−1^ were estimated for *Delftia* sp*.* isolate UME58 (0.39 ± 0.05 h^−1^), *Serratia* sp. isolate UMA615 (0.38 ± 0.05 h^−1^), *Agrobacterium* sp. isolate UMA610 (0.37 ± 0.02 h^−1^), *Achromobacter* sp. isolate UMC71 (0.33 ± 0.04 h^−1^), *Cupriavidus* sp. isolate UME74 (0.29 ± 0.04 h^−1^), *Variovorax* sp. isolate UMC13 (0.25 ± 0.01 h^−1^), *Sphingobacterium* sp. isolate UME9 (0.23 ± 0.02 h^−1^), and *Burkholderia* sp. isolate UMG646 (0.15 ± 0.07 h^−1^).

Moreover, the characteristic acidification of the culture medium reported for strain M1, during growth using β-myrcene as sole carbon-source (Santos and Sá-Correia 2009; Soares-Castro et al. 2017) was detected for isolates clustered with this strain (UMA601, UMA603, UMA643, UMC76, UMC631, UMC3103, UMC3106, UMC3129, and UME83), as well as for the *Pseudomonas* sp. isolates UMC65 and UME65 (Table S3).

### The β-myrcene biotransformation of several soil isolates produced β-myrcene derivatives previously reported in strain M1

A panel of 17 Myr^+^ isolates was selected to identify the diversity of β-myrcene derivatives produced in biotransformation experiments, by GC–MS. The panel comprised representatives of different species, to which the β-myrcene supplementation supported considerable growth and biomass production (OD_600 nm_ > 1.0): *Achromobacter* sp. isolate UMC46; *Cupriavidus* sp. isolate UME77; *Delftia* sp. isolate UME58; *Pseudomonas* sp. UMC76, UMC631, UMC3103, UMC3106, UMC3129, UME83, UMA601, UMA603, UMA643, UME65, and UME3145; *Serratia* sp. isolate UME734; *Sphingobacterium* sp. isolate UME9; and *Variovorax* sp. isolate UMC13 (Table S3).

The analysis of culture’s supernatants of the strain M1 detected all key β-myrcene derivatives reported previously in the biotransformation of the monoterpene substrate (Table 2; Soares-Castro et al. 2017): myrcen-8-ol (C_10_H_16_O; compound 4), myrcenal (C_10_H_14_O; compound 3; (2E)-2-methyl-6-methylideneocta-2,7-dien-1-al), myrcenoic acid (C_10_H_14_O_2_; compound 5; (2E)-2-methyl-6-methylideneocta-2,7-dienoic acid), 4-methyl-3-hexenoic acid (C_7_H_12_O_2_; 4-Me-3-HA; compound 2), and 4-methylhexanoic acid (C_7_H_14_O_2_; 4-MeHA; compound 1).

**Table 2:** Metabolites successfully identified in β-myrcene (C_10_H_16_) biotransformation experiments with the selected representatives of the panel of Myr^+^ soil isolates, which registered considerable growth and biomass production (OD600 nm > 1.0) in β-myrcene-supplemented cultures. The *Pseudomonas* sp. strain M1 was used as reference

Metabolite identification						
*Predicted metabolite ID* *(formula) (compound no.)*		4-MeHA C_7_H_14_O_2_ (1)	4-Me-3-HA C_7_H_12_O_2_ (2)	Myrcenal C_10_H_14_O (3)	Myrcen-8-ol C_10_H_16_O (4)	Myrcenoic acid C_10_H_14_O_2_ (5)
*ID method*		MS, RI	MS, RI	MS	MS, RI	MS
*Retention index*		1039	1079	1217	1241	1393
GC peaks^a^						
*Reference strain*	M1	0.004 ± 2.8E − 4	0.077 ± 3.6E − 2	0.081 ± 9.8E − 3	0.215 ± 2.6E − 2	0.560 ± 1.0E − 1
*Pseudomonas* spp.	UMA601	0.003	0.037 ± 6.0E − 3	0.071 ± 2.0E − 2	0.282 ± 3.5E − 2	0.355 ± 6.2E − 2
*(M1-like)*	UMA603	0.004 ± 2.3E − 5	0.123 ± 1.1E − 2	*ND*	0.032	1.041 ± 1.3E − 2
	UMA643	0.003	0.032 ± 3.7E − 3	0.098	0.312	0.180 ± 3.1E − 2
	UMC3103	0.013 ± 3.4E − 3	0.308 ± 1.3E − 1	0.034	0.125	0.506 ± 2.9E − 1
	UMC3106	0.003 ± 3.4E − 4	0.048 ± 9.1E − 3	0.020 ± 2.8E − 3	0.036 ± 2.6E − 2	0.222 ± 2.1E − 2
	UMC3129	0.010 ± 1.9E − 3	0.092 ± 1.6E − 2	0.056 ± 6.2E − 3	0.425 ± 1.1E − 1	1.179 ± 1.2E − 1
	UMC631	0.020 ± 7.2E − 3	0.202 ± 4.1E − 2	*ND*	0.070	0.349 ± 4.4E − 2
	UMC76	0.002 ± 2.5E − 4	0.050 ± 2.6E − 2	0.025 ± 7.1E − 3	0.162 ± 1.7E − 2	0.874 ± 3.0E − 1
	UME83	0.004	0.057 ± 3.9E − 2	*ND*	0.053	0.366 ± 1.2E − 1
*Pseudomonas* sp.	UME65	0.018 ± 7.2E − 3	0.436 ± 7.4E − 2	0.071 ± 1.9E − 2	0.183 ± 6.5E − 2	9.821 ± 2.3
*Sphingobacterium* sp.	UME9	0.012 ± 4.2E − 3	0.324 ± 8.5E − 2	*ND*	0.022 ± 8.9E − 3	11.027 ± 4.8
*Variovorax* sp.	UMC13	0.008 ± 1.3E − 3	0.171 ± 6.5E − 3	*ND*	0.016	1.201 ± 2.1E − 1
*Cupriavidus* sp.	UME77	*ND*	*ND*	*ND*	0.002 ± 1.0E − 3	*ND*

^**a**^Peak areas of the metabolites identified in the supernatants of each strain, normalized as a percentage of the total area of the chromatogram, shown as average values ± standard deviation when replicates were available, at mid-exponential phase of β-myrcene (7-methyl-3-methylideneocta-1,6-diene) biotransformation. *4-MeHA*, 4-Methylhexanoic acid; *4-Me-3-HA*, 4-Methyl-3-hexenoic acid; *Myrcenal* ((2E)-2-methyl-6-methylideneocta-2,7-dien-1-al); *Myrcen-8-ol* ((2E)-2-methyl-6-methylideneocta-2,7-dien-1-ol); *Myrcenoic acid* ((2E)-2-methyl-6-methylideneocta-2,7-dienoic acid); *MS*, mass spectrum search; *RI*, retention index; *ND*, not detected in any replicate

Significantly, myrcen-8-ol (compound 4), myrcenoic acid (compound 5), 4-methyl-3-hexenoic acid (compound 2) and 4-methylhexanoic acid (compound 1) were also detected in supernatants of the *Pseudomonas* sp. isolates UMC76, UMC631, UMC3103, UMC3106, UMC3129, UME83, UMA601, UMA603, UMA643, and UME65, as well as in supernatants of the *Sphingobacterium* sp. isolate UME9 and *Variovorax* sp. isolate UMC13. The initial oxidation product of β-myrcene biotransformation, myrcen-8-ol (compound 4), was also detected in cultures of *Cupriavidus* sp. isolate UME77.

Moreover, we were not able to identify β-myrcene derivatives, including any of the metabolites reported in the literature, in the analysis of the supernatants of *Achromobacter* sp. isolate UMC46, *Delftia* sp. isolate UME58, *Pseudomonas* sp. isolate UME3145, and *Serratia* sp. isolate UME734.

### Whole-genome sequencing of 14 bacteria harboring the Myr^+^ trait

The isolates *Cupriavidus* sp. UME77; *Pseudomonas* sp. UMC65, UME65, UMA601, UMA603, UMA643, UMC76, UMC631, UMC3103, UMC3106, UMC3129, and UME83; *Sphingobacterium* sp. UME9; and *Variovorax* sp. UMC13, for which β-myrcene derivatives were successfully identified, were selected for whole-genome sequencing and characterization of the respective genomic background underlying the Myr^+^ phenotype. Even though the draft genomes were deposited in 2020, the assemblies were confirmed with recent versions of the tools, without significative changes in the outcome (contiguity, size, etc.). Assembly metrics of the final draft genomes are shown in Table S3.

**Fig. 2:**
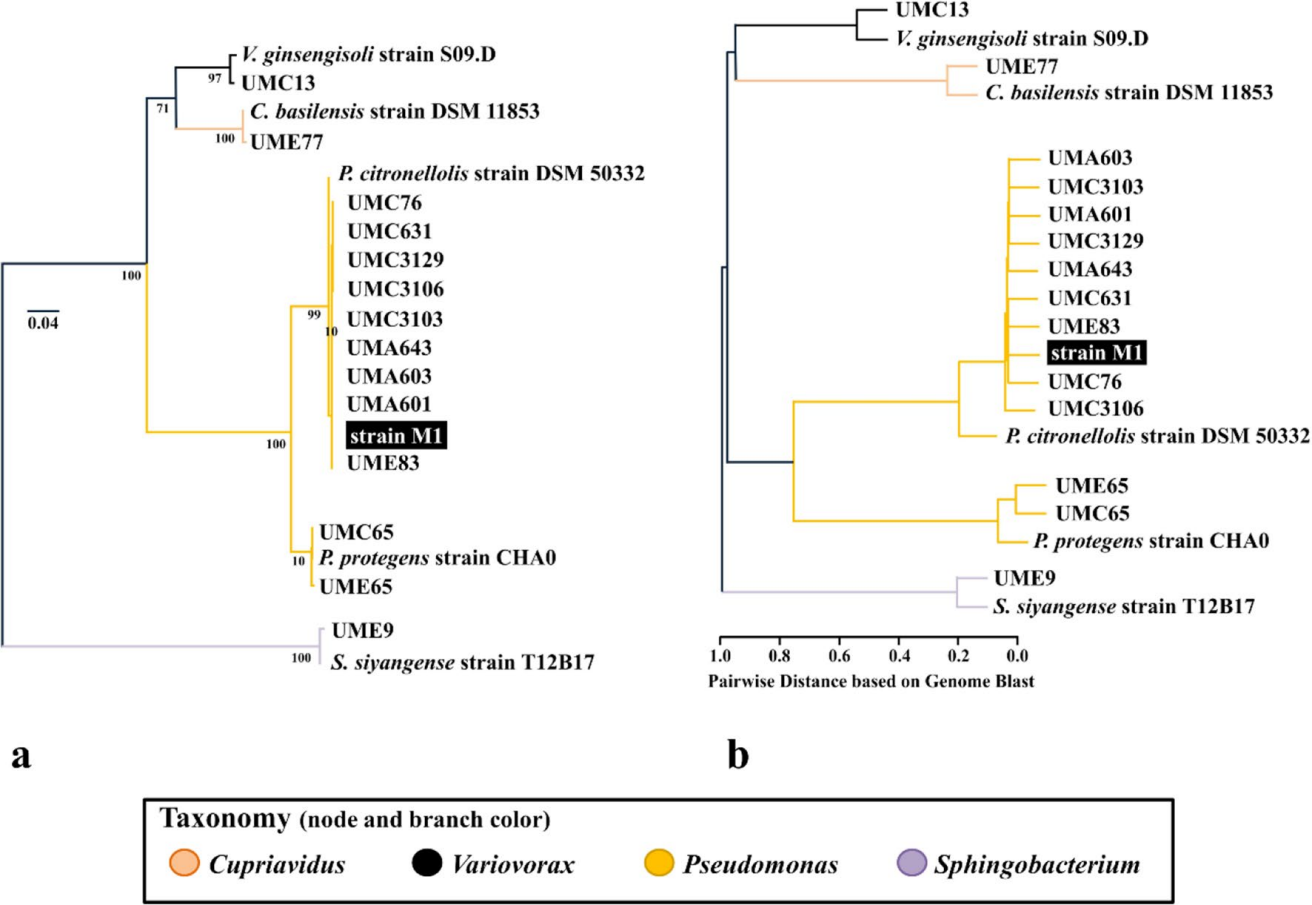
Phylogeny of the 14 Myr^+^ isolates and phylogenetically closer reference strains. Panel **a** shows the phylogenetic tree with midpoint rooting based on the alignment of the 16S rRNA gene sequence extracted from the assembled and reference genomes. Bootstrap values presented as percentage are shown in the tree branches. Panel **b** shows the hierarchical clustering based on the pairwise distances estimated with BLAST alignments between all strains. Two identical genomes would result in a distance value of 0. The distances were estimated according to the ratio of nucleotide identity per total length of the compared genomes. Branches are colored according to the bacterial genera. Isolate codes: “UMC,” Cabril; “UME,” Ermida; “UMA,” Guimarães; “UMG,” Grândola. The estimated ANI values are available in Table S4

The estimation of the average nucleotide identity (ANI) against reference genomes deposit in NCBI allowed to determine phylogenetic closer strains with available sequenced genomes, for the validation of the taxonomic assignment and the following comparative genomics analysis. The phylogenetic relationships between Myr^+^ bacteria and the selected references were assessed by the 16S rRNA gene alignment (Fig. 2a) and validated by whole-genome pairwise BLAST-based alignment (Fig. 2b).

The draft genome of the isolates *Cupriavidus* sp. UME77 showed 96.7% of average nucleotide identity (ANI) against the genome of *Cupriavidus basilensis* strain DSM 11853, whereas the draft genomes of *Sphingobacterium* sp. UME9 and *Variovorax* sp. UMC13 showed 97.7% and 85.9% to the genomes of the closest references *Sphingobacterium paramultivorum* strain BIGb0170 and *Variovorax ginsengisoli* strain S09.D, respectively (Table S4). Moreover, the pairwise genomic distances also evidenced a high degree of genome similarity between several isolates retrieved from different locations, such as the pair UMC65 and UME65, which showed 98.9% of ANI to the type strain *Pseudomonas protegens* CHA0 (Fig. 2b and Table S4). These 2 isolates shared 99.9% of nucleotide identity among them, even though their sampling source was separated by 8.5 km. The isolates UMA601, UMA603, UMA643, UMC76, UMC631, UMC3103, UMC3106, UMC3129, and UME83, also retrieved from 3 different locations, shared around 98.1% of ANI with the *Pseudomonas citronellolis* and around 99.9% of ANI with the strain M1 (GenBank accession CP094343). Having the high percentage of ANI similarity, we hypothesize that M1 strain (and related isolates) belongs to *Pseudomonas citronellolis* species. This hypothesis is corroborated by a genome-based taxonomy comparative analysis using TYGS (https://tygs.dsmz.de/) which indicated that *Pseudomonas* sp. M1 and related isolates belong to *Pseudomonas citronellolis* species. However, further experimental evidences are required to confirm such hypothesis.

Even though β-myrcene biotransformations with *Cupriavidus* sp. UME77, *Sphingobacterium* sp. UME9, and *Variovorax* sp. UMC13 generated β-myrcene-derivatives reported in strain M1, the genome mining of their 3 genomes did not detect close homologs of the gene products of the M1 28-kb β-myrcene core-code (BLASTP analysis showed < 70% identity at the protein level, whose hits were not organized in genetic clusters; Table S6). The genome analysis of these 3 isolates, thereby, hinted the existence of alternative functional modules underlying their Myr^+^ trait and the detected metabolic profile. On the contrary, the activation of the reporter system in the isolates UMA601, UMA603, UMA643, UMC76, UMC631, UMC3103, UMC3106, UMC3129, UME83, UMC65, and UME65 (Table S3), together with the similar metabolic footprint of β-myrcene-derivatives as reported for strain M1, suggested that their genomes might harbor the full or partial 28-kb locus responsible for the M1 Myr^+^ trait.

### The 28-kb β-myrcene core-code is located within a 76-kb genomic *locus* conserved in strain M1 and 11 other *Pseudomonas* isolates

**Fig. 3:**
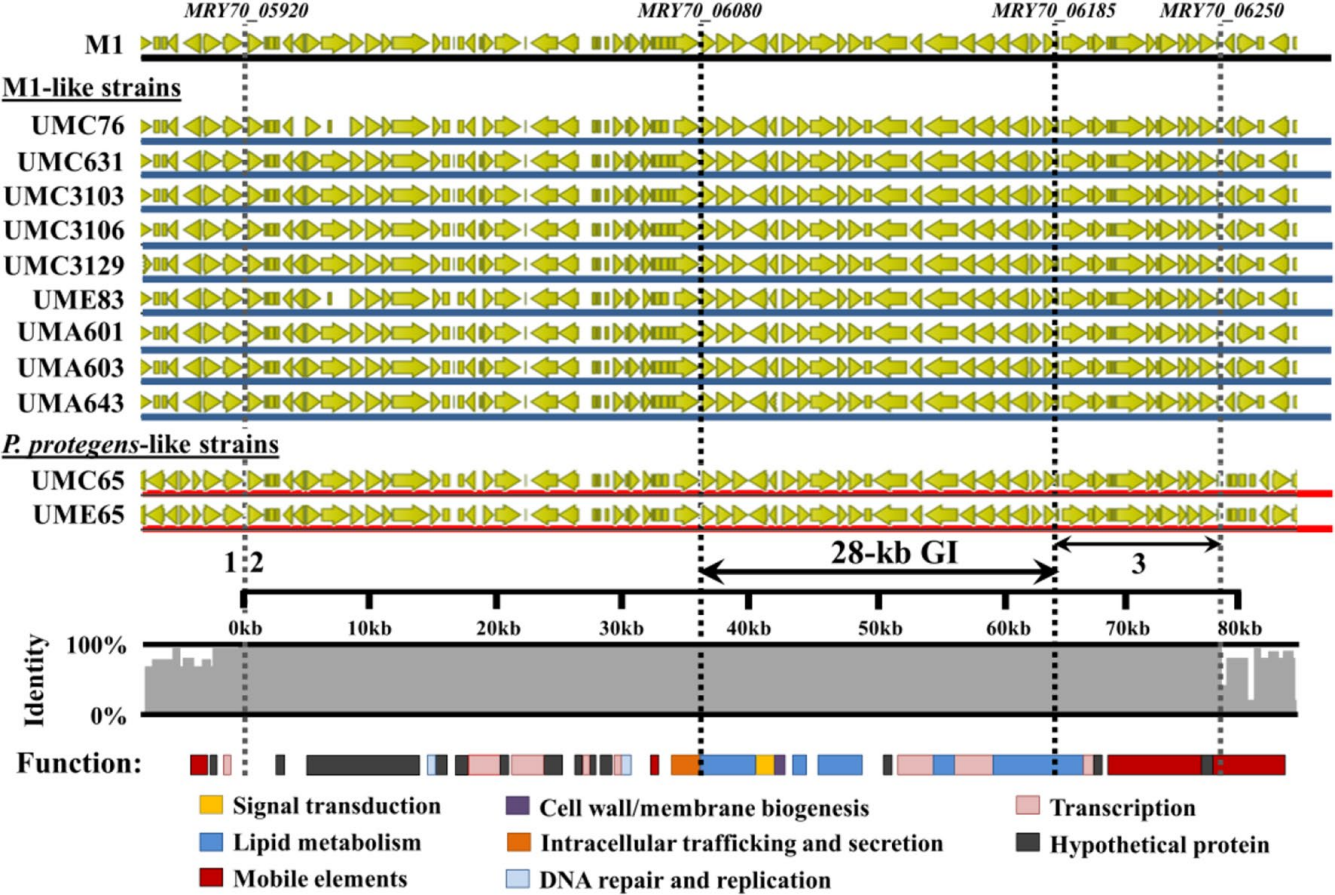
Nucleotide conservation of a 76-kb genomic region among 11 *Pseudomonas* spp., comprising the β-myrcene-responsive *locus* characterized in *Pseudomonas* sp. M1. The DNA backbone sequence is colored in blue for M1-like isolates and in red for *P. protegens*-like isolates: M1, *Pseudomonas* sp. M1 (*MRY70_05920-MRY70_06250*); UMC76 (*A9979_06855-A9979_07180*); UMC631 (*FCN13_00270-FCN13_00620*); UMC3103 (*FCN10_11380-FCN10_11730*); UMC3106 (*FCJ51_00270-FCJ51_00620*); UMC3129 (*FCN12_00270-FCN12_00620*); UME83 (*A9972_23140-A9972_23470*); UMA601 (*FCJ53_19215*-*FCJ53_19415* and *FCJ53_19910-FCJ53_20055*); UMA603 (*FCJ55_16630-FCJ55_16830* and *FCJ55_04610-FCJ55_04755*); UMA643 (*FCJ56_13360-FCJ56_13560* and *FCJ56_04410-FCJ56_04555*); UMC65 (*A9978_04360-A9978_04690*); UME65 (*A9971_23150-A9971_23480*). The identity per base of the nucleotide alignment is represented in the grey plot. The grey vertical dotted lines depict the flanks of the genomic 76-kb *locus* fully conserved in the five *Pseudomonas* strains, whereas the black vertical dotted lines depict the flanks of the β-myrcene 28-kb core-code in *Pseudomonas* sp. M1. CDS within this 76-kb conserved *locus* are colored by function, based on COG classes. The category “Mobile elements” includes integrases, transposases, conjugative elements and plasmid-associated proteins. Using the genome of M1 strain as reference, the “1” represents the local of insertion downstream the GMP synthase (*guaA*) gene *MRY70_05915* and corresponding homologs; “2” indicates the upstream flank starting with the *MRY70_05920* gene, which codes for a putative integrase; “3” represents the downstream flank, ending with a cluster coding putative conjugal transfer proteins from Tra and Trb family (*MRY70_06190-MRY70_06250*)

A closer inspection of the draft genomes of *Pseudomonas sp.* isolates UMC76, UMC631, UMC3103, UMC3106, UMC3129, UME83, UMA601, UMA603, and UMA643 revealed the presence of the 22 genes of the 28-kb GI from strain M1 (*MRY70_06080-MRY70_06185*), showing 100% nucleotide identity and alignment coverage (Table S5), displaying a similar structural organization, in the 9 M1-like *Pseudomonas* isolates (Fig. 3), as supported by the GC–MS analysis and the screening carried out the reporter system.

Furthermore, the comparative genomics analysis between the strain M1 and the other sequenced *Pseudomonas* isolates evidenced the presence of the fully conserved 28-kb in the *P. protegens*-like isolates UMC65 and UME65. Strikingly, a whole-genome alignment between these *Pseudomonas* sp. isolates showed that the full nucleotide conservation of the β-myrcene core-code was not restricted to the 28-kb GI and included, in fact, a larger genomic region of approximately 76-kb (Fig. 3).

This 76-kb conserved region was located downstream the housekeeping gene *guaA* (*MRY70_05915*), coding for a glutamine-hydrolyzing GMP synthase, in the 12 aligned genomes (Fig. 3, indicated with “1”), flanked by a gene coding for a putative integrase (*MRY70_05920* in strain M1; Fig. 3, indicated with “2”) and a cluster coding putative conjugal transfer proteins (*MRY70_06190- MRY70_06250*) in strain M1; Fig. 3, indicated with “3”). The integrase MRY70_05920 is homologous to the tyrosine site-specific P4 integrase of *P. mendocina* ymp located in the genomic island ympGI^*guaA*^ (Pmen_3484: 91.6% a.a. identity and 99% of sequence coverage) and of *Acidovorax avenae* subsp. *citrulli* AAC00-1 located in the genomic island AAC00-1GI^guaA^ (Aave_3366: 85% a.a. identity covering 99% of aligned sequence) strains (Table S6). The conjugational cluster located downstream of the β-myrcene core-code encodes a putative *trbBCDEJLFGI* operon and a *traG* gene, described as homologous to the conjugation system of IncP1 plasmids (RP4/Ti-like) (Lessl et al. 1992). Although homology for several genes located in the upstream flank of the 76-kb locus was not found in the ICEberg database, the conjugational cluster showed a.a. identity ranging from 67 to 89% with the Tn4371 ICE from *Cupriavidus oxalatica* A5 and from 37 to 89% with the ICE-KKS from *Acidovorax* sp. KKS102 (Table S6), both belonging to a family of ICEs (the Tn4371 family) only reported in *Gamma-proteobacteria* and *Beta-proteobacteria* (Springael et al. 2001; Toussaint et al. 2003). Besides the β-myrcene 28-kb catabolic modules, genes comprised within the integrative *locus* code for putative proteins involved in DNA integration and replication, transcription, and a major fraction encode proteins with unknown function (Fig. 3).

## Discussion

The enrichment protocol and culture-dependent approach used in the present study allowed to isolate 150 β-myrcene-biotransforming bacteria living in the rhizosphere of cork oak trees and eucalyptus trees, from 4 different geographic sources in Portugal: from Cabril, Ermida, and Guimarães in the north, as well as from Grândola in the south. The ability to biotransform β-myrcene was, thus, widespread in the sampled soil communities of cork oak and eucalyptus forests. Hence, the soil surrounding these forests may comprise a valuable reservoir of potential β-myrcene (and other monoterpenes) catalysts.

The library of isolates extended the current taxonomy associated with the aerobic β-myrcene catabolism to 15 bacterial genera, ubiquitous in the environment and reported to establish biological interactions with plants. In fact, members of the genera *Achromobacter*, *Acinetobacter*, *Burkholderia*, *Delftia*, *Enterobacter*, *Pseudomonas*, *Raoultella*, *Serratia*, *Sphingobacterium* and *Variovorax* reported in this study were also found in the soil microbial communities of *Q. suber* (Bevivino et al. 2014). *Pseudomonas* spp. were also detected in the rhizosphere of several eucalyptus trees (Berendsen et al. 2015; da Silva Fonseca et al. 2018). Furthermore, the analysis of the microbiome from the pinewood nematode *Bursaphelenchus xylophilus* resulted in the identification of genes putatively associated with the catabolism of α-pinene, limonene, and geraniol, in *Pseudomonas*, *Achromobacter*, and *Agrobacterium* (Cheng et al. 2013). Similarly, the microbiome of bark beetles seems to comprise monoterpene-metabolizing bacteria from the genera *Pseudomonas*, *Rahnella*, *Serratia*, and *Stenotrophomonas* (Adams et al. 2011). In 2014, the metatranscriptome analysis of the resin-tolerant microbial community associated with pine tree galls, formed by the moth *Retinia resinella*, detected expression of genes associated with α-pinene catabolism and the acyclic terpene utilization pathway (*atu* pathway), widespread in more than 40 bacterial genera, from which *Pseudomonas* was the most abundant bacterial genus (Vilanova et al. 2014). Although functional characterization is required to assess the catalytic novelty of the reported genes, these metagenomics studies support the taxonomic knowledge acquired in the present study associating a broader taxonomic context and genomic background with the monoterpene-biotransforming trait.

The growth kinetics of the isolated bacteria was evaluated with representative isolates belonging to the different genera (Fig. 1; Table S3), in cultures supplemented with β-myrcene as carbon source. All assayed isolates phylogenetically closer to our reference *Pseudomonas* sp. strain M1 reached OD_600 nm_ values higher than 1.5 at 24 h of growth and μ_myr_ higher than 0.7 h^−1^ (with the exception of UMA601). This outstanding catabolic performance of the M1-like isolates was paralleled by other *Pseudomonas* spp. from the *P. putida* clade (strains UMG604, UMG605 and UMG612 from clade G11 in Fig. S2, with μ_myr_ > 0.80 h^−1^), the *Citrobacter* isolate UMG736 (0.85 ± 0.02 h^−1^) and the *Raoultella* isolate UMG700 (0.70 ± 0.02 h^−1^), despite registering lower biomass yield. To our knowledge, *Achromobacter* spp., *Acinetobacter* spp., *Burkholderia* spp., *Lelliottia* spp., *Raoultella* spp., and *Sphingobacterium* spp. have never been experimentally associated to the ability of catalyzing catabolic transformations in the monoterpene backbone, whereas some strains of several *Pseudomonas* species (e.g., *P. putida*, *P. aeruginosa*, *P. citronellolis*, *P. protegens*, among others), *Serratia marcescens*, and *Citrobacter braakii* have been studied by their monoterpene-biotransforming enzymatic repertoire (Soares-Castro et al. 2021, and references therein).

The detection of several β-myrcene derivatives, previously reported for the β-myrcene catabolism of strain M1, such as myrcen-8-ol, myrcenoic acid, 4-methyl-3-hexenoic acid and 4-methylhexanoic acid, in *Pseudomonas* sp. isolates UMC76, UMC631, UMC3103, UMC3106, UMC3129, UME83, UMA601, UMA603, UMA643, and UME65, evidenced that their β-myrcene biotransformation pathways follow similar enzymatic steps, towards the channeling of the β-myrcene derivatives to the central metabolism. Additionally, the GFP fluorescence of the M1 β-myrcene hydroxylase MyrH promoter-probe was only induced by the supplementation of β-myrcene in M1-like isolates and in the *P. protegens*-like isolates UMC65 and UME65, thereby indicating the presence of M1-GI related modules in the genome of these 11 *Pseudomonas* spp. Evidence of the dissemination of the 28-kb GI in the isolates UMC76, UMC631, UMC3103, UMC3106, UMC3129, UME83, UMA601, UMA603, UMA643, UMC65, and UME65 was obtained by high-throughput genome sequencing, which revealed the full nucleotide and structural conservation of the 28-kb β-myrcene core code found in strain M1 (Fig. 3). Most importantly, the complete conservation of the nucleotide sequence was extended to a region spanning 76-kb, whose genetic content resembled the *guaA*-associated ICEs (Song et al. 2012).

As self-transmissible elements, ICEs encode the machinery required to control their excision and mediate conjugation (Wozniak and Waldor 2010). In M1 cells grown in the presence of β-myrcene, gene expression levels quantified by RNA-seq showed the induction of several CDS associated with the mobility of integrative and conjugative elements (Soares-Castro and Santos 2015), namely the upregulation of the integrase-coding gene (*MRY70_05920*) by 39-fold, the putative transcriptional regulator of the AlpA family (*MRY70_05935*) by fourfold, and the *MRY70_06190-MRY70_06250* cluster, putatively coding conjugative transfer Tra and Trb proteins, by levels ranging from 1.7- to 3.5-fold. In particular, the putative β-myrcene-induced AlpA regulator (98% identity and 99% of alignment coverage with the putative AlpA-family regulator from *P. stutzeri*; accession WP_044316124) is reported to be the positive regulatory factor of P4 integrases (Kirby et al. 1994; Trempy et al. 1994). The induction of these CDS suggested the presence of an active regulatory cascade targeting the mobilization systems (or remnants of them) upon β-myrcene supplementation. These activation mechanisms could result in the excision of the 76-kb *locus*, albeit experimental evidences of a free excised circular replicon were never obtained. Such mobilization events may have resulted in the acquisition of the 76-kb ICE by the *P. protegens*-like strains UMC65 and UME65, since M1-like bacteria were also isolated in both Cabril (UMC76, UMC631, UMC3103, UMC3106, UMC3129) and Ermida (UME83) soils. Evidence of the existence of multiple replicons in strain M1 was not detected during assemblies with different types of datasets. The transference frequency and ability to transfer a mobile genetic element, such as genomic islands, even between closely related strains, have been described to be dependent on the host background (Minoia et al. 2008; Miyazaki et al. 2012): in part resulting from the integration of the GI-borne regulatory system within the cell global regulatory network and potential cross-talks between conserved regulatory signals of different mobile elements co-existing in the host, which can lead to the repression or induction of the island. Furthermore, the genetic structure and nucleotide composition of the 76-kb *locus* containing the β-myrcene core-code was conserved in all 12 strains of our library, even though they were isolated from geographically and chronologically distinct sources. *Pseudomonas* sp. strain M1 was isolated from sediments of the Rhine river (Netherlands) in 1997–1998, *Pseudomonas* sp. strains UMC76, UME83, UMC65, and UME65 were isolated from soil rhizosphere collected in 2015, and *Pseudomonas* sp. strains UMC631, UMC3103, UMC3106, UMC3129, UMA601, UMA603, and UMA643 were isolated from soil rhizosphere collected in 2017, from forests in the north of Portugal. Therefore, following experimental evidence is required to understand if the 76-kb locus containing the β-myrcene core-code is stably integrated in the genome or being actively mobilized, even if at low frequencies in the population, as described for the ICEclc in *P. knackmussii* strain B13 (Minoia et al. 2008; Miyazaki et al. 2012). Following work will also be focused on the characterization of factors modulating a potential excision (e.g., growth phase, efficiency of excision, and conjugation), putative interplay between the catabolic modules and the mobility-related elements, and the range of hosts, currently only observed in *Pseudomonas* spp.

The comparative genomics between *Pseudomonas* sp. strain M1 and the newly sequenced *Pseudomonas* sp. strains UMC76, UMC631, UMC3103, UMC3106, UMC3129, UME83, UMA601, UMA603, and UMA643 showed 99.9% of genomic similarity, according to the estimated ANI (Table S4). The high similarity detected at the genomic level between the 10 strains may suggest that they belong to the same species, resulting from unique evolutionary track of each strain, probably driven by niche-specific circumstances. Strikingly, the full conservation of the 76-kb *locus* hints the existence of molecular mechanisms maintaining the integrity and stability of this catabolic *locus*, which may exert a tight control of its transcription (e.g., DNA methylation associated to DNA repair systems) (Camacho and Casadesús 2002; Casadesús and Low 2006). Although we did not find clear evidence in the literature linking DNA methylation patterns to the conservation of specific DNA sequences and bacteria evolution, several reports associate N6-adenine methylation through Dam methyltransferase with the effectiveness of DNA repair systems. In *Escherichia coli* strain K12, mutants of the Dam methyltransferase resulted in an increase spontaneous mutation frequency up to 46-fold when compared to the Dam^+^ strain (Marinus and Morris 1975). Similar effects were also described for Dam^–^ mutants on *Serratia typhimurium* (Torreblanca and Casadesús 1996). Moreover, the review of Wion and Casadesús (2006) reported that Dam-mediated methylation can also repress the mobility of the bacterial transposons IS10, IS50, Tn5, and Tn903 in *Escherichia coli*. Dam-mediated methylation of GATC sites in the promoter of both transposons hampered the binding of RNA polymerase and inhibited the transcription of the transposase required for transposition to occur. Additionally, methylation of GATC sites at the inner terminus of both IS10 and IS50 transposons inhibited the transposase activity, thus contributing to the host genome stability (Wion and Casadesús 2006).

Furthermore, the performed physiological, molecular, and analytical screenings suggested that the other Myr^+^ bacteria did not harbor a genomic context compatible with the activation of the promoter of the β-myrcene hydroxylase MyrH of the strain M1. The genomes of the sequenced isolates not harboring the 76-kb ICE (*Sphingobacterium* sp. isolate UME9, *Variovorax* sp. isolate UMC13, and *Cupriavidus* sp. isolate UME77) were further screened in silico for homologs of known monoterpene-catabolizing enzymes, involved in (i) the *atu* pathway from *Pseudomonas citronellolis* (gene products from *atuRABCDEFGH)* (Förster-Fromme et al. 2006); (ii) oxidation of linallool/geraniol by *Castellaniella defragrans* 65Phen (GeoA, GeoB and Ldi) (Lüddeke et al. 2012) and *Pseudomonas putida* (LinC) (Ropp et al. 1993); (iii) catabolism of limonene and carveol by *Rhodococcus erythropolis* DCL14 (LimABC and Mlhb) (van der Werf et al. 1999; van der Werf and Boot 2000) and *Geobacillus stearothermophilus* BR388 (gene product of pOT435 gene) (Cheong and Oriel 2000); (iv) catabolism of camphor by *Pseudomonas putida* NCIMB10007 (gene products of *camABCDEFG*) (Poulos et al. 1985; Bell et al. 2010) and *Rhodococcus* sp. (*camK*) (Roberts et al. 2004); (v) catabolism of *p*-cymene by *P. putida* F1 (gene products of *cymBCAaAbDE* and *cmtABCDEFGHI*) (Eaton 1996, 1997); and (vi) oxidation of 1,8-cineole by *Citrobacter braakii* (CinA) (Hawkes et al. 2002). This BLASTP analysis did not detect close homologs of the queried enzymes (BLASTP showed < 70% identity at the protein level, not organized in an operon-like structure; Table S5), and, thereby, candidate clusters coding for putative β-myrcene-biotransforming modules could not be considered. Having this in mind, the production of myrcen-8-ol, myrcenoic acid, 4-methyl-3-hexenoic acid, and 4-methylhexanoic acid by *Sphingobacterium* sp. isolate UME9 and *Variovorax* sp. isolate UMC13 supported the existence of β-myrcene-biotransforming modules with similar catalytic properties to those found in strain M1, even though corresponding to a distinct Myr^+^ genetic trait. The detection of myrcen-8-ol in β-myrcene-supplemented cultures of *Cupriavidus* sp. isolate UME77 suggested, at least, a common initial oxidation step with strain M1, but also derived from a different, and yet to be defined, Myr^+^ genetic trait. On the contrary, the lack of identification of known β-myrcene derivatives (or other monoterpene-related isomers, alcohols, aldehydes, or acids) in β-myrcene-growing cultures of *Achromobacter* sp. UMC46, *Delftia* sp. UME58, *Pseudomonas* sp. UME3145, and *Serratia* sp. UME734 may have derived from the fact that standard metabolite databases do not comprise a large number of mass spectra of β-myrcene-derived compounds (Soares-Castro et al. 2017), suggesting different metabolites may be generated by novel catabolic machinery. This also reflects the lack of knowledge available regarding β-myrcene catabolic pathways, which in turn impairs the successful metabolite identification (Soares-Castro et al. 2021). The missed detection of the initial β-myrcene derivatives could also have been influenced by strain-specific physiological traits (e.g., the rate of biotransformation) coupled to the single time-point analysis carried out at mid-exponential phase, even though saturating amounts of the monoterpene were used to supplement the cultures and ensure an unlimited feed of the substrate in a aqueous-gaseous equilibrium.

Nevertheless, the Myr^+^ genetic trait underlying the β-myrcene catabolism in the isolates not harboring the M1-related ICE remains to be identified and validated with functional approaches (e.g., RNA-seq) in following work, which will greatly increase the knowledge regarding the β-myrcene biotransformation in these strains.

## Supplementary Information

Below is the link to the electronic supplementary material.

Supplementary file1 (PDF 1319 KB)

## Data Availability

The Whole Genome Shotgun projects generated in this work have been deposited at DDBJ/EMBL/GenBank under the following accession numbers: *Cupriavidus* sp. strain UME77 (LZZU00000000), *Pseudomonas* sp. strain UMC65 (LZZX00000000), *Pseudomonas* sp. strain UME65 (LZZQ00000000), *Pseudomonas* sp. strain UMC76 (LZZY00000000), *Pseudomonas* sp. strain UMC631 (SUQJ00000000), *Pseudomonas* sp. strain UMC3103 (SWJU00000000), *Pseudomonas* sp. strain UMC3106 (SUQK00000000), *Pseudomonas* sp. strain UMC3129 (SWJT00000000), *Pseudomonas* sp. strain UME83 (LZZR00000000), *Pseudomonas* sp. strain UMA601 (SUQI00000000), *Pseudomonas* sp*.* strain UMA603 (SUQS00000000), *Pseudomonas* sp. strain UMA643 (SUQL00000000), *Sphingobacterium* sp. strain UME9 (LZZP00000000), *Variovorax* sp. strain UMC13 (LZZW00000000).

## Strain availability

The reference strain, *Pseudomonas* sp. M1, and the representative isolates harboring the myr + *Pseudomonas* sp. UMA601, *Pseudomonas* sp. UMAC76, *Pseudomonas* sp. UME83 have been deposited at the University of Coimbra Bacteria Culture Collection (UCCCB) under the accession numbers UCCCB 159, UCCCB 160, UCCCB 161, and UCCCB 162, respectively, and at the Spanish Type Culture Collection (CECT) under the accession numbers CECT 20780, CECT 20781, CECT 20782, and CECT 20783, respectively.
