# Supplementary material for: Bioprospection of the bacterial β-myrcene-biotransforming trait in the rhizosphere
Source: Appl Microbiol Biotechnol. 2023 Jul 5;107(16):5209–24. doi: 10.1007/s00253-023-12650-w (PMC10386936; doi:10.1007/s00253-023-12650-w)
Supplement: Supplementary file 1 — Supplementary file1 (PDF 1319 KB) [file 253_2023_12650_MOESM1_ESM.pdf]

SUPPLEMENTARY MATERIALS

for

Applied Microbiology and Biotechnology

Bioprospection of the bacterial β-myrcene-biotransforming trait in the rhizosphere

Pedro Soares-Castro,[a]* Filipa Soares,[a] Francisca Reis,[a] Teresa Lino-Neto,[a] and Pedro M. Santos[a]#

[a] CBMA – Centre of Molecular and Environmental Biology, University of Minho, Campus de Gualtar, Braga, Portugal.

#Address correspondence to Pedro M. Santos, psantos@bio.uminho.pt.

* Present address: Pedro Soares-Castro, Instituto de Medicina Molecular, Faculdade de Medicina, Universidade de Lisboa, Av. Prof. Egas Moniz, Lisbon, Portugal.

Supplementary Tables

Table S1. Geographic and environmental features of the sampling sites. Averages of annual precipitation (Pannual) and annual temperature (Tannual) over the past 30 years (1987 – 2017). Soil tillage indicates the degree of anthropogenic-related disturbance derived from agricultural exploitation. The Mediterranean climate classification was determined by calculating the Emberger index parameter (Q), which takes into account the annual precipitation (Pannual) and maximal (Tmax) and minimal (Tmin) temperatures of the hottest and coldest months, respectively, during the sampling year, where Q = 100 x Pannual (Tmax2 - Tmin2). This information was obtained from the Portuguese Sea and Atmosphere Institute (IPMA).

	**Cabril**	**Ermida**	**Grândola**	**Guimarães**
**Coordinates**	41°45'43.05"N 8°1'39.09"W (41.76194444, -8.02750000)	41°42´39.76"N 8°6´14.87"W (41.71111111, -8.10416667)	38°11´32.37"N 8°37´11.41"W (38.19222222, -8.61972222)	41°27'46.24"N 8°16'2.92"W (41.46277778, -8.26750000)
**Altitude (m)**	492	627	150	267
**pH**	4.9 (strongly acid)	5.1 (strongly acid)	5.5 (slightly acid)	6.1 (slightly acid)
**Precipitation (Pannual - mm)**	1448.4	1448.4	735.6	1141
**Temperature (Tannual - ºC)**	12.7	12.7	16.6	15.1
**Emberger index (*Q*)[a]**	186.6 (humid)	186.6 (humid)	77.5 (semi-arid)	176,0 (humid)
**Soil tillage**	non-tilled/wild forest	non-tilled/wild forest	tilled/ Pasture and cork exploration	non-tilled/wild forest
**Soil texture**	sand	sand	Loamy sand	Loamy sand

Table S2. Total of 150 soil isolates obtained from the enrichment cultures with β-myrcene selection and the corresponding metadata regarding the source of isolation. Clade code represents the location of the isolate in the phylogenetic tree of Fig. S1 and S2: Sphingobacteriia – S1; Alphaproteobacteria – A1; Betaproteobacteria – B1-B5; Gammaproteobacteria – G1-G13. Sequences of 16S rRNA gene obtained by Sanger were identified by using the Ribosomal Database Project (RDP). Score indicates the pairwise sequence identity of the alignment (1 = 100 %)

Isolate ID		Physiology data upon isolation		Taxonomy	
	Source	OD 24h	pH 24h	Clade	Best hit (type strains); Accession no.
Achromobacter sp.					
UMC46	Cabril	0.88	7.00	B1	*Achromobacter spanius (T); LMG 5911; AY170848*
UMC71	Cabril	1.34	6.90	B1	*Achromobacter mucicolens (T); R-46658; HE613446*
UME14	Ermida	2.14	7.0	B1	*Achromobacter spanius (T); LMG 5911; AY170848*
UMG3138	Grândola	0.75	6.9	B1	*Achromobacter spanius (T); LMG 5911; AY170848*
Acinetobacter sp.					
UMG648	Grândola	0.72	7.1	G9	*Acinetobacter radioresistens (T); DSM 6976; X81666*
Agrobacterium sp.					
UMA610	Guimarães	0.69	6.9	A1	*Agrobacterium radiobacter (T); IAM 12048; AB247615*
UMA611	Guimarães	0.56	7.1	A1	*Agrobacterium radiobacter (T); IAM 12048; AB247615*
Burkholderia sp.					
UMC728	Cabril	0.54	6.8	B4	*Burkholderia caledonica (T); LMG 19076; AF215704*
UMC610	Cabril	1.44	6.8	B4	*Burkholderia diffusa (T); R-15930; AM747629*
UMC708	Cabril	1.00	6.7	B4	*Burkholderia metallica (T); R-16017; AM747632*
UMG617	Grândola	1.06	6.9	B4	*Burkholderia lata (T); 383; CP000150*
UMG646	Grândola	0.79	6.8	B4	*Burkholderia metallica (T); R-16017; AM747632*
Citrobacter sp.					
UMG732	Grândola	0.75	6.8	G4	*Citrobacter gillenii (T); CDC 4693-86; AF025367*
UMG736	Grândola	0.87	6.8	G4	*Citrobacter gillenii (T); CDC 4693-86; AF025367*
Cupriavidus sp.					
UMC53	Cabril	0.88	7.0	B5	*Cupriavidus basilensis (T); DSM 11853; AF312022*
UMC54	Cabril	1.19	6.9	B5	*Cupriavidus basilensis (T); DSM 11853; AF312022*
UMC58	Cabril	0.87	6.9	B5	*Cupriavidus basilensis (T); DSM 11853; AF312022*
UMC63	Cabril	0.65	6.9	B5	*Cupriavidus basilensis (T); DSM 11853; AF312022*
UME27	Ermida	1.26	7.0	B5	*Cupriavidus basilensis (T); DSM 11853; AF312022*
UME38	Ermida	1.46	7.0	B5	*Cupriavidus basilensis (T); DSM 11853; AF312022*
UME52	Ermida	0.53	6.9	B5	*Cupriavidus basilensis (T); DSM 11853; AF312022*
UME61	Ermida	0.89	7.0	B5	*Cupriavidus basilensis (T); DSM 11853; AF312022*
UME74	Ermida	0.62	6.9	B5	*Cupriavidus basilensis (T); DSM 11853; AF312022*
UME77	Ermida	1.32	7.0	B5	*Cupriavidus basilensis (T); DSM 11853; AF312022*
UME79	Ermida	0.78	7.0	B5	*Cupriavidus basilensis (T); DSM 11853; AF312022*
Delftia sp.					
UME55	Ermida	0.57	7.0	B3	*Delftia lacustris (T); 332; EU888308*
UME58	Ermida	1.02	6.9	B3	*Delftia lacustris (T); 332; EU888308*

Enterobacter sp.					
UMC73	Cabril	0.44	6.9	G6	*Enterobacter ludwigii (T); DSMZ 16688; AJ853891*
UME64	Ermida	0.48	7.0	G6	*Enterobacter ludwigii (T); DSMZ 16688; AJ853891*
UMG600	Grândola	0.50	6.9	G6	*Enterobacter ludwigii (T); DSMZ 16688; AJ853891*
UMG601	Grândola	0.52	7.1	G6	*Enterobacter ludwigii (T); DSMZ 16688; AJ853891*
UMA3112	Guimarães	0.74	6.9	G6	*Enterobacter asburiae (T); JCM6051; AB004744*
UMA3113	Guimarães	0.55	7.0	G6	*Enterobacter asburiae (T); JCM6051; AB004744*
UMA3115	Guimarães	0.70	6.9	G6	*Enterobacter asburiae (T); JCM6051; AB004744*
UMA3122	Guimarães	0.51	6.9	G6	*Enterobacter ludwigii (T); DSMZ 16688; AJ853891*
UMA3124	Guimarães	0.56	6.8	G6	*Enterobacter asburiae (T); JCM6051; AB004744*
UMA3133	Guimarães	0.56	7.0	G6	*Enterobacter asburiae (T); JCM6051; AB004744*
Lelliottia sp.					
UMG3140	Grândola	1.19	6.8	G5	*Lelliottia amnigena (T); JCM1237; AB004749*
UMG3147	Grândola	1.00	6.8	G5	*Lelliottia amnigena (T); JCM1237; AB004749*
UMA3111	Guimarães	0.75	6.8	G5	*Lelliottia amnigena; E63; HQ407240*
UMA3120	Guimarães	0.77	6.7	G5	*Lelliottia amnigena (T); JCM1237; AB004749*
UMA3121	Guimarães	0.68	6.7	G5	*Lelliottia amnigena (T); JCM1237; AB004749*
UMA3123	Guimarães	0.65	6.7	G5	*Lelliottia amnigena (T); JCM1237; AB004749*
Pseudomonas sp.					
UMA631	Guimarães	0.52	7.0	G10	*Pseudomonas gessardii (T); CIP 105469; AF074384*
UMA606	Guimarães	0.50	7.0	G10	*Pseudomonas helmanticensis (T); OHA11; HG940537*
UMG3100	Grândola	0.61	6.8	G10	*Pseudomonas koreensis (T); Ps 9-14; AF468452*
UMG3125	Grândola	0.77	6.8	G10	*Pseudomonas koreensis (T); Ps 9-14; AF468452*
UMA638	Guimarães	0.50	6.9	G10	*Pseudomonas lurida (T); DSM 15835; AJ581999*
UME3100	Ermida	1.73	6.9	G11	*Pseudomonas putida (T); IAM 1236; D84020*
UME3101	Ermida	1.62	7.0	G11	*Pseudomonas putida (T); IAM 1236; D84020*
UME3107	Ermida	1.45	6.9	G11	*Pseudomonas putida (T); IAM 1236; D84020*
UME3114	Ermida	1.43	6.9	G11	*Pseudomonas putida (T); IAM 1236; D84020*
UME3119	Ermida	1.20	6.9	G11	*Pseudomonas putida (T); IAM 1236; D84020*
UME3136	Ermida	1.27	7.0	G11	*Pseudomonas putida (T); IAM 1236; D84020*
UME3140	Ermida	1.44	6.9	G11	*Pseudomonas putida (T); IAM 1236; D84020*
UME3142	Ermida	0.74	6.8	G11	*Pseudomonas putida (T); IAM 1236; D84020*
UME3145	Ermida	1.45	6.9	G11	*Pseudomonas putida (T); IAM 1236; D84020*
UME3146	Ermida	1.46	6.9	G11	*Pseudomonas putida (T); IAM 1236; D84020*
UMG602	Grândola	1.05	6.9	G11	*Pseudomonas putida (T); IAM 1236; D84020*
UMG604	Grândola	0.85	6.9	G11	*Pseudomonas putida (T); IAM 1236; D84020*
UMG605	Grândola	1.28	6.9	G11	*Pseudomonas putida (T); IAM 1236; D84020*
UMG606	Grândola	1.09	6.9	G11	*Pseudomonas putida (T); IAM 1236; D84020*
UMG609	Grândola	1.14	6.9	G11	*Pseudomonas putida (T); IAM 1236; D84020*
UMG610	Grândola	0.89	6.8	G11	*Pseudomonas putida (T); IAM 1236; D84020*
UMG611	Grândola	1.30	6.9	G11	*Pseudomonas putida (T); IAM 1236; D84020*
UMG612	Grândola	1.29	6.9	G11	*Pseudomonas putida (T); IAM 1236; D84020*
UMG622	Grândola	1.20	6.9	G11	*Pseudomonas putida (T); IAM 1236; D84020*
UMA648	Guimarães	0.50	6.9	G11	*Pseudomonas yamanorum (T); 8H1; EU557337*
UMC1	Cabril	0.65	7.0	G12	*Pseudomonas protegens (T); CHA0; AJ278812*
UMC12	Cabril	0.53	7.0	G12	*Pseudomonas protegens (T); CHA0; AJ278812*

UMC29	Cabril	0.56	7.0	G12	*Pseudomonas protegens (T); CHA0; AJ278812*
UMC56	Cabril	1.56	6.5	G12	*Pseudomonas protegens (T); CHA0; AJ278812*
UMC65	Cabril	1.55	6.4	G12	*Pseudomonas protegens (T); CHA0; AJ278812*
UME1	Ermida	0.57	7.0	G12	*Pseudomonas protegens (T); CHA0; AJ278812*
UME63	Ermida	1.37	6.4	G12	*Pseudomonas protegens (T); CHA0; AJ278812*
UME65	Ermida	1.43	6.5	G12	*Pseudomonas protegens (T); CHA0; AJ278812*
UME72	Ermida	1.31	6.5	G12	*Pseudomonas protegens (T); CHA0; AJ278812*
UMG3110	Grândola	0.82	6.8	G12	*Pseudomonas protegens (T); CHA0; AJ278812*
UMG3128	Grândola	0.76	7.0	G12	*Pseudomonas protegens (T); CHA0; AJ278812*
UMG3145	Grândola	0.85	6.8	G12	*Pseudomonas protegens (T); CHA0; AJ278812*
UMC76	Cabril	1.89	6.2	G13	*Pseudomonas delhiensis (T); RLD-1; DQ339153*
UMC617	Cabril	1.66	6.0	G13	*Pseudomonas delhiensis (T); RLD-1; DQ339153*
UMC630	Cabril	1.70	6.1	G13	*Pseudomonas delhiensis (T); RLD-1; DQ339153*
UMC631	Cabril	1.90	5.9	G13	*Pseudomonas delhiensis (T); RLD-1; DQ339153*
UMC3102	Cabril	1.69	6.0	G13	*Pseudomonas delhiensis (T); RLD-1; DQ339153*
UMC3103	Cabril	2.04	5.9	G13	*Pseudomonas delhiensis (T); RLD-1; DQ339153*
UMC3104	Cabril	1.78	6.0	G13	*Pseudomonas delhiensis (T); RLD-1; DQ339153*
UMC3105	Cabril	1.96	6.0	G13	*Pseudomonas delhiensis (T); RLD-1; DQ339153*
UMC3106	Cabril	2.26	5.8	G13	*Pseudomonas delhiensis (T); RLD-1; DQ339153*
UMC3108	Cabril	1.54	6.1	G13	*Pseudomonas delhiensis (T); RLD-1; DQ339153*
UMC3109	Cabril	1.59	6.0	G13	*Pseudomonas delhiensis (T); RLD-1; DQ339153*
UMC3110	Cabril	1.92	5.8	G13	*Pseudomonas delhiensis (T); RLD-1; DQ339153*
UMC3112	Cabril	1.53	6.0	G13	*Pseudomonas delhiensis (T); RLD-1; DQ339153*
UMC3113	Cabril	1.93	5.9	G13	*Pseudomonas delhiensis (T); RLD-1; DQ339153*
UMC3114	Cabril	1.42	6.1	G13	*Pseudomonas delhiensis (T); RLD-1; DQ339153*
UMC3122	Cabril	1.82	5.9	G13	*Pseudomonas delhiensis (T); RLD-1; DQ339153*
UMC3129	Cabril	1.65	6.2	G13	*Pseudomonas delhiensis (T); RLD-1; DQ339153*
UME28	Ermida	1.27	6.1	G13	*Pseudomonas delhiensis (T); RLD-1; DQ339153*
UME34	Ermida	1.31	6.2	G13	*Pseudomonas delhiensis (T); RLD-1; DQ339153*
UME76	Ermida	1.64	6.1	G13	*Pseudomonas delhiensis (T); RLD-1; DQ339153*
UME83	Ermida	1.80	6.1	G13	*Pseudomonas delhiensis (T); RLD-1; DQ339153*
UMA601	Guimarães	1.11	6.1	G13	*Pseudomonas delhiensis (T); RLD-1; DQ339153*
UMA603	Guimarães	1.25	6.1	G13	*Pseudomonas citronellolis (T); DSM 50332T; Z76659*
UMA604	Guimarães	1.20	6.2	G13	*Pseudomonas citronellolis (T); DSM 50332T; Z76659*
UMA643	Guimarães	1.44	6.1	G13	*Pseudomonas citronellolis (T); DSM 50332T; Z76659*
UMA647	Guimarães	1.07	6.3	G13	*Pseudomonas citronellolis (T); DSM 50332T; Z76659*
Raoultella sp.					
UMG700	Grândola	0.85	6.9	G7	*Raoultella terrigena (T); ATCC33257T; Y17658*
UMG701	Grândola	0.81	6.9	G7	*Raoultella terrigena (T); ATCC33257T; Y17658*
UMG707	Grândola	0.85	6.9	G7	*Raoultella ornithinolytica (T); CIP 103364; U78182*
Serratia sp.					
UMA609	Guimarães	0.50	7.0	G1	*Serratia fonticola (T); DSM 4576; AJ233429*
UMA615	Guimarães	0.50	6.9	G1	*Serratia fonticola (T); C1; FJ790328*
UMA621	Guimarães	0.50	7.0	G1	*Serratia fonticola (T); DSM 4576; AJ233429*
UMA3110	Guimarães	0.72	6.9	G1	*Serratia fonticola (T); DSM 4576; AJ233429*
UMA3114	Guimarães	0.72	6.9	G1	*Serratia fonticola (T); DSM 4576; AJ233429*

UMA3116	Guimarães	0.80	6.7	G1	*Serratia fonticola (T); DSM 4576; AJ233429*
UMA3131	Guimarães	0.71	6.9	G1	*Serratia fonticola (T); DSM 4576; AJ233429*
UMA3132	Guimarães	0.69	7.0	G1	*Serratia fonticola (T); DSM 4576; AJ233429*
UMA3134	Guimarães	0.73	6.9	G1	*Serratia fonticola (T); DSM 4576; AJ233429*
UMA3135	Guimarães	0.69	6.9	G1	*Serratia fonticola (T); DSM 4576; AJ233429*
UMA3136	Guimarães	0.64	7.0	G1	*Serratia fonticola (T); DSM 4576; AJ233429*
UMA3130	Guimarães	0.67	6.8	G1	*Serratia quinivorans (T); DSM 4597; AJ233435*
UMG706	Grândola	0.71	6.9	G3	*Serratia nematodiphila (T); DZ0503SBS1; EU036987*
UMG709	Grândola	0.66	6.9	G3	*Serratia marcescens (T); KRED; AB061685*
UMC601	Cabril	0.53	6.8	G2	*Serratia plymuthica (T); DSM 4540; AJ233433*
UMC3100	Cabril	0.50	6.8	G2	*Serratia plymuthica (T); DSM 4540; AJ233433*
UMG634	Grândola	0.56	6.9	G2	*Serratia plymuthica (T); DSM 4540; AJ233433*
UMG647	Grândola	0.74	6.9	G2	*Serratia plymuthica (T); DSM 4540; AJ233433*
UME603	Ermida	0.87	6.8	G2	*Serratia proteamaculans (T); DSM 4543; AJ233434*
UME606	Ermida	0.86	6.8	G2	*Serratia proteamaculans (T); DSM 4543; AJ233434*
UME607	Ermida	0.62	6.8	G2	*Serratia proteamaculans (T); DSM 4543; AJ233434*
UME616	Ermida	0.64	6.8	G2	*Serratia proteamaculans (T); DSM 4543; AJ233434*
UME619	Ermida	0.78	6.8	G2	*Serratia proteamaculans (T); DSM 4543; AJ233434*
UME629	Ermida	0.50	7.0	G2	*Serratia proteamaculans (T); DSM 4543; AJ233434*
UME701	Ermida	0.83	6.8	G2	*Serratia proteamaculans (T); DSM 4543; AJ233434*
UME708	Ermida	0.69	6.8	G2	*Serratia proteamaculans (T); DSM 4543; AJ233434*
UME710	Ermida	0.77	6.9	G2	*Serratia proteamaculans (T); DSM 4543; AJ233434*
UME715	Ermida	0.75	6.8	G2	*Serratia proteamaculans (T); DSM 4543; AJ233434*
UME718	Ermida	0.51	6.8	G2	*Serratia proteamaculans (T); DSM 4543; AJ233434*
UME731	Ermida	0.72	6.9	G2	*Serratia proteamaculans (T); DSM 4543; AJ233434*
UME734	Ermida	0.57	6.8	G2	*Serratia proteamaculans (T); DSM 4543; AJ233434*
Shigella sp.					
UMG705	Grândola	0.91	6.9	G8	*Shigella flexneri (T); X96963*
UMG725	Grândola	0.83	6.9	G8	*Shigella flexneri (T); X96963*
UMG619	Grândola	0.69	6.9	G8	*Shigella sonnei (T); type strain: CECT 4887; FR870445*
UMG741	Grândola	0.72	6.8	G8	*Shigella sonnei (T); type strain: CECT 4887; FR870445*
Sphingobacterium sp.					
UMC8	Cabril	0.56	7.0	S1	*Sphingobacterium siyangense (T); SY1; EU046272*
UMC42	Cabril	0.51	7.0	S1	*Sphingobacterium siyangense (T); SY1; EU046272*
UME9	Ermida	0.68	6.9	S1	*Sphingobacterium siyangense (T); SY1; EU046272*
UME29	Ermida	0.50	6.9	S1	*Sphingobacterium siyangense (T); SY1; EU046272*
Variovorax sp.					
UMC13	Cabril	0.86	7.0	B2	*Variovorax boronicumulans (T); BAM-48; AB300597*
UMC48	Cabril	0.50	7.0	B2	*Variovorax boronicumulans (T); BAM-48; AB300597*

Table S3. Total of 43 Myr+ isolates selected for physiological characterization regarding their growth kinetics parameters registered from cultures growing with β-myrcene as carbon source (μmyr, maximum specific growth rate; OD600 nm, optical density registered at 8 h and 24 h of growth; pH values registered at 8 h and 24 h of growth). The column "Reporter P5-GFP Fold induction" shows the ratio between the fluorescence of the reporter system in the presence of β-myrcene and the fluorescence without supplementation of the monoterpene (a value around 1 means that there was no difference in the induction of the reporter with and without the monoterpene. The columns "WGS sequencing project" correspond to the basic parameters of the draft assemblies.

Isolate ID	Physiology data from cultures grown with β-myrcene as carbon source					Reporter P5-GFP Fold induction	WGS sequencing project		
	μ_{myr}	$OD_{600\ nm}$ (8h)	$OD_{600\ nm}$ (24h)	pH (8h)	pH (24h)		Genbank accession number	No. contigs	Estimated genome size
Achromobacter sp.									
**UMC46**	0.46±0.04	0.87±0.04	1.79±0.08	7.00±0.03	7.02±0.03	-	-	-	-
**UMC71**	0.33±0.04	1.05±0.17	1.38±0.10	6.97±0.01	6.96±0.04	-	-	-	-
Acinetobacter sp.									
**UMG648**	0.62±0.06	0.58±0.13	0.50±0.14	6.83±0.02	7.04±0.03	-	-	-	-
Agrobacterium sp.									
**UMA610**	0.37±0.02	0.80±0.11	0.80±0.01	6.67±0.03	6.65±0.07	-	-	-	-
Burkholderia sp.									
**UMC610**	0.45±0.02	0.51±0.01	0.46±0.01	6.93±0.07	6.89±0.01	-	-	-	-
**UMC708**	0.45±0.02	0.55±0.06	0.94±0.02	6.76±0.04	6.90±0.03	-	-	-	-
**UMG646**	0.15±0.07	0.22±0.04	0.53±0.02	7.01±0.01	6.92±0.01	-	-	-	-
Citrobacter sp.									
**UMG736**	0.85±0.02	0.92±0.17	0.86±0.06	6.75±0.10	6.80±0.02	-	-	-	-
Cupriavidus sp.									
**UME74**	0.29±0.04	0.35±0.01	0.52±0.02	6.92±0.00	6.90±0.01	-	-	-	-
**UME77**	0.51±0.08	0.74±0.01	1.00±0.03	7.08±0.01	7.08±0.04	-	LZZU00000000	98	8.3 Mbp
Delftia sp.									
**UME58**	0.39±0.05	0.92±0.26	1.41±0.24	6.98±0.01	7.01±0.01	-	-	-	-
Lelliottia sp.									
**UMG3140**	0.53±0.02	0.61±0.01	0.61±0.01	6.85±0.04	6.96±0.02	-	-	-	-
**UMA3121**	0.60±0.01	0.75±0.14	0.72±0.17	6.93±0.02	6.95±0.02	-	-	-	-
Pseudomonas sp.									
**UMG3125**	0.56±0.05	0.58±0.01	0.47±0.04	6.90±0.01	6.89±0.03	-	-	-	-
**UME3100**	0.62±0.12	0.91±0.01	1.41±0.01	6.86±0.01	6.91±0.01	-	-	-	-
**UME3119**	0.58±0.02	0.69±0.01	1.07±0.11	6.90±0.01	6.94±0.02	-	-	-	-
**UME3142**	0.49±0.01	0.59±0.07	0.54±0.02	6.95±0.00	6.96±0.00	-	-	-	-
**UME3145**	0.66±0.13	1.12±0.01	1.20±0.04	6.88±0.00	6.90±0.01	-	-	-	-
**UMG604**	0.82±0.02	0.52±0.02	0.52±0.02	6.97±0.01	7.00±0.02	-	-	-	-
**UMG605**	0.89±0.14	0.79±0.14	0.80±0.13	6.89±0.00	6.86±0.02	-	-	-	-
**UMG610**	0.49±0.01	1.00±0.01	1.36±0.01	6.89±0.01	6.95±0.01	-	-	-	-
**UMG612**	0.82±0.07	0.99±0.13	0.95±0.06	6.97±0.00	6.98±0.00	-	-	-	-
**UMG622**	0.40±0.01	0.60±0.00	0.35±0.05	6.97±0.03	7.00±0.04	-	-	-	-
**UMC65**	0.43±0.04	1.38±0.18	1.30±0.05	6.39±0.04	6.35±0.07	2.6±0.01	LZZX00000000	25	7.2 Mbp
**UME65**	0.51±0.01	1.23±0.04	1.35±0.11	6.45±0.01	6.43±0.04	2.0±0.05	LZZQ00000000	21	7.1 Mbp

**UMG3145**	0.51±0.03	0.51±0.04	0.42±0.03	6.90±0.02	6.88±0.06	-	-	-	-
**UMC76**	0.88±0.03	1.52±0.01	1.92±0.18	6.33±0.01	6.29±0.10	3.5±0.13	LZZY00000000	105	7.0 Mbp
**UMC631**	0.77±0.01	1.51±0.01	1.67±0.16	6.20±0.22	6.26±0.19	2.3±0.02	SUQJ00000000	122	6.9 Mbp
**UMC3103**	0.81±0.04	2.13±0.04	1.62±0.05	6.03±0.02	6.26±0.04	3.2±0.21	SWJU00000000	127	6.9 Mbp
**UMC3106**	0.72±0.05	1.75±0.01	1.67±0.15	6.30±0.05	6.25±0.07	2.8±0.17	SUQK00000000	95	6.7 Mbp
**UMC3129**	0.95±0.01	1.45±0.01	1.75±0.09	6.13±0.04	5.99±0.01	4.7±0.02	SWJT00000000	85	6.9 Mbp
**UME83**	0.73±0.03	1.27±0.09	1.54±0.20	6.37±0.03	6.17±0.24	2.3±0.16	LZZR00000000	70	7.0 Mbp
**UMA601**	0.65±0.05	1.73±0.05	1.79±0.04	5.90±0.15	5.97±0.07	2.0±0.01	SUQI00000000	101	6.9 Mbp
**UMA603**	0.98±0.09	1.40±0.16	1.58±0.11	6.07±0.01	5.92±0.35	3.0±0.01	SUQS00000000	105	6.9 Mbp
**UMA643**	0.78±0.03	1.59±0.11	2.14±0.06	6.05±0.06	5.45±0.13	2.5±0.14	SUQL00000000	124	6.9 Mbp
Raoultella sp.									
**UMG700**	0.70±0.02	0.72±0.04	0.46±0.00	6.84±0.01	7.00±0.01	-	-	-	-
Serratia sp.									
**UME603**	0.56±0.09	0.58±0.04	0.58±0.02	6.93±0.00	6.95±0.00	-	-	-	-
**UME734**	0.44±0.02	1.01±0.02	0.23±0.01	6.96±0.00	7.00±0.01	-	-	-	-
**UMA615**	0.38±0.05	0.62±0.04	0.69±0.06	6.82±0.06	6.91±0.04	-	-	-	-
**UMA3116**	0.53±0.02	0.60±0.03	0.60±0.01	6.92±0.07	6.86±0.04	-	-	-	-
Shigella sp.									
**UMG705**	0.61±0.06	0.72±0.07	0.68±0.04	6.89±0.00	6.85±0.02	-	-	-	-
Sphingobacterium sp.									
**UME9**	0.23±0.02	1.15±0.18	1.66±0.15	6.72±0.05	6.91±0.09	-	LZZP00000000	15	6.8 Mbp
Variovorax sp.									
**UMC13**	0.25±0.01	1.13±0.17	1.48±0.13	6.96±0.01	7.00±0.05	-	LZZW00000000	43	7.1 Mbp

Table S4. Whole-genome similarity between the 14 Myr+ isolates and phylogenetically closer reference strains, determined by the Average Nucleotide Identity (ANI)

Gamma-Proteobacteria			
	***Pseudomonas protegens* strain CHA0**	***Pseudomonas sp.* strain M1**	***Pseudomonas citronellolis* strain DSM50332**
Pseudomonas spp.			
UMC65	98.9	85.1	84.3
UME65	98.9	85.1	84.3
UMC76	84.3	99.9	98.2
UMC631	84.3	99.9	98.2
UMC3103	84.3	99.9	98.2
UMC3106	84.3	99.9	98.2
UMC3129	84.3	99.9	98.2
UME83	84.3	99.9	98.2
UMA601	84.3	99.9	98.2
UMA603	84.3	99.9	98.2
UMA643	84.3	99.9	98.2
Beta-Proteobacteria			
	***Cupriavidus basilensis* strain DSM 11853**	***Variovorax ginsengisoli* strain S09.D**	
Cupriavidus sp.			
UME77	96.7	82.2	
Variovorax sp.			
UMC13	83.6	85.9	
Sphingobacteriia			
	***Sphingobacterium paramultivorum* strain BIGb0170**		
Sphingobacterium sp.			
UME9	97.7		

Table S5. Protein homology of gene products coded by the 28-kb genomic island from M1 strain, with genetic background of strains isolated from soil enrichment cultures. BLASTP search was performed using the predicted proteomes of each soil-derived strain as subject sequences (custom database). ID, Identity between query protein sequence and blast hit. PM1, *Pseudomonas sp.* strain M1; *Cupriavidus sp.* isolate UME77; *Pseudomonas sp.* isolate UMC631; *Pseudomonas sp.* isolate UMC3103; *Pseudomonas sp.* isolate UMC3106; *Pseudomonas sp.* isolate UMC3129; *Pseudomonas sp.* isolate UMA601; *Pseudomonas sp.* isolate UMA603; *Pseudomonas sp.* isolate UMA643; PC76, *Pseudomonas sp.* isolate UMC76; *Pseudomonas sp.* isolate UME83; *Pseudomonas sp.* isolate UMC65; *Pseudomonas sp.* isolate UME65; *Sphingobacterium sp.* isolate UME83; *Variovorax sp.* isolate UMC13.

Strain	Best subject hit (locus_tag)	E-value	Alignment Length	no. gaps	ID (%)	no. mismatch	Coverage (%)
**MRY70_06080: Putative butyryl-CoA dehydrogenase**							
PM1	MRY70_06080	0	378	0	100	0	100
UME77	A9975_07925	0	375	0	67	123	99
UMA601	FCJ53_19235	0	378	0	100	0	100
UMA603	FCJ55_16650	0	378	0	100	0	100
UMA643	FCJ56_13380	0	378	0	100	0	100
UMC631	FCN13_00450	0	378	0	100	0	100
UMC3103	FCN10_11560	0	378	0	100	0	100
UMC3106	FCJ51_00450	0	378	0	100	0	100
UMC3129	FCN12_00450	0	378	0	100	0	100
UMC76	A9979_07010	0	378	0	100	0	100
UME83	A9972_23310	0	378	0	100	0	100
UMC65	A9978_04530	0	378	0	100	0	100
UME65	A9971_23320	0	378	0	100	0	100
UME9	A9970_19110	2.00E-117	374	1	47	198	99
UMC13	A9977_23820	3.00E-171	375	0	62	143	99
**Strain**	**Best subject hit (locus_tag)**	**E-value**	**Alignment Length**	**no. gaps**	**ID (%)**	**no. mismatch**	**Coverage (%)**
**MRY70_06085: Putative acyl-CoA dehydrogenase**							
M1	MRY70_06085	0	406	0	100	0	100
CE77	A9975_07925	2.00E-50	390	20	33	241	95
UMA601	FCJ53_19230	0	406	0	100	0	100
UMA603	FCJ55_16645	0	406	0	100	0	100
UMA643	FCJ56_13375	0	406	0	100	0	100
UMC631	FCN13_00455	0	406	0	100	0	100
UMC3103	FCN10_11565	0	406	0	100	0	100

UMC3106	FCJ51_00455	0	406	0	100	0	100
UMC3129	FCN12_00455	0	406	0	100	0	100
UMC76	A9979_07010	0	406	0	100	0	100
UME83	A9972_23310	0	406	0	100	0	100
UMC65	A9978_04525	0	406	0	100	0	100
UME65	A9971_23315	0	406	0	100	0	100
SE9	A9970_19110	2.00E-58	390	23	37	224	94
VC13	A9977_23820	3.00E-51	391	22	32	242	95
**Strain**	**Best subject hit (locus_tag)**	**E-value**	**Alignment Length**	**no. gaps**	**ID (%)**	**no. mismatch**	**Coverage (%)**
**MRY70_06090: Putative 3-ketoacyl-CoA thiolase**							
M1	MRY70_06090	0	396	0	100	0	100
CE77	A9975_07915	2.00E-175	397	2	62	148	99
UMA601	FCJ53_19225	0	396	0	100	0	100
UMA603	FCJ55_16640	0	396	0	100	0	100
UMA643	FCJ56_13370	0	396	0	100	0	100
UMC631	FCN13_00460	0	396	0	100	0	100
UMC3103	FCN10_11570	0	396	0	100	0	100
UMC3106	FCJ51_00460	0	396	0	100	0	100
UMC3129	FCN12_00460	0	396	0	100	0	100
UMC76	A9979_07010	0	378	0	100	0	100
UME83	A9972_23310	0	378	0	100	0	100
UMC65	A9978_04520	0	396	0	100	0	100
UME65	A9971_23310	0	396	0	100	0	100
SE9	A9970_25630	2.00E-107	394	4	45	213	99
VC13	A9977_23830	3.00E-160	396	4	59	159	99
**Strain**	**Best subject hit (locus_tag)**	**E-value**	**Alignment Length**	**no. gaps**	**ID (%)**	**no. mismatch**	**Coverage (%)**
**MRY70_06095: Putative histidine kinase chemotaxis sensory transducer**							
M1	MRY70_06095	0	491	0	100	0	100
CE77	A9975_14425	8.00E-35	352	32	32	207	71
UMA601	FCJ53_19220	0	491	0	100	0	100
UMA603	FCJ55_16635	0	491	0	100	0	100
UMA643	FCJ56_13365	0	491	0	100	0	100
UMC631	FCN13_00465	0	491	0	100	0	100
UMC3103	FCN10_11575	0	491	0	100	0	100
UMC3106	FCJ51_00465	0	491	0	100	0	100
UMC3129	FCN12_00465	0	491	0	100	0	100
UMC76	A9979_07010	0	491	0	100	0	100
UME83	A9972_23310	0	491	0	100	0	100
UMC65	A9978_04515	0	491	0	100	0	100

UME65	A9971_23305	0	491	0	100	0	100
SE9	A9970_09535	2	26	0	42	15	5
VC13	A9977_14785	3.00E-24	362	20	29	238	72
**Strain**	**Best subject hit (locus_tag)**	**E-value**	**Alignment Length**	**no. gaps**	**ID (%)**	**no. mismatch**	**Coverage (%)**
**MRY70_06100: Putative OmpW family protein**							
M1	MRY70_06100	0	167	0	100	0	100
CE77	A9975_22315	1.00E-12	152	14	30	93	90
UMA601	FCJ53_19215	0	167	0	100	0	100
UMA603	FCJ55_16630	0	167	0	100	0	100
UMA643	FCJ56_13360	0	167	0	100	0	100
UMC631	FCN13_00470	0	167	0	100	0	100
UMC3103	FCN10_11580	0	167	0	100	0	100
UMC3106	FCJ51_00470	0	167	0	100	0	100
UMC3129	FCN12_00470	0	167	0	100	0	100
UMC76	A9979_07010	0	167	0	100	0	100
UME83	A9972_23310	0	167	0	100	0	100
UMC65	A9978_04510	0	167	0	100	0	100
UME65	A9971_23300	0	167	0	100	0	100
SE9	A9970_10440	2.00E-12	161	12	29	102	89
VC13	A9977_22805	7.00E-10	143	6	29	95	82
**Strain**	**Best subject hit (locus_tag)**	**E-value**	**Alignment Length**	**no. gaps**	**ID (%)**	**no. mismatch**	**Coverage (%)**
**MRY70_06105: Putative acetyl-CoA acetyltransferase**							
M1	MRY70_06105	0	392	0	100	0	100
CE77	A9975_00005	0	392	0	73	104	99
UMA601	FCJ53_20055	0	392	0	100	0	100
UMA603	FCJ55_04755	0	392	0	100	0	100
UMA643	FCJ56_04410	0	392	0	100	0	100
UMC631	FCN13_00475	0	392	0	100	0	100
UMC3103	FCN10_11585	0	392	0	100	0	100
UMC3106	FCJ51_00475	0	392	0	100	0	100
UMC3129	FCN12_00475	0	392	0	100	0	100
UMC76	A9979_07010	0	392	0	100	0	100
UME83	A9972_23310	0	392	0	100	0	100
UMC65	A9978_04505	0	392	0	100	0	100
UME65	A9971_23295	0	392	0	100	0	100
SE9	A9970_10345	3.00E-80	398	17	38	230	99
VC13	A9977_15715	0	392	0	73	103	99
**Strain**	**Best subject hit (locus_tag)**	**E-value**	**Alignment Length**	**no. gaps**	**ID (%)**	**no. mismatch**	**Coverage (%)**

**MRY70_06110: Putative 2-nitropropane dioxygenase**							
M1	MRY70_06110	0	324	0	100	0	100
CE77	A9975_00015	1.00E-177	322	0	72	91	99
UMA601	FCJ53_20050	0	324	0	100	0	100
UMA603	FCJ55_04750	0	324	0	100	0	100
UMA643	FCJ56_04415	0	324	0	100	0	100
UMC631	FCN13_00480	0	324	0	100	0	100
UMC3103	FCN10_11590	0	324	0	100	0	100
UMC3106	FCJ51_00480	0	324	0	100	0	100
UMC3129	FCN12_00480	0	324	0	100	0	100
UMC76	A9979_07010	0	324	0	100	0	100
UME83	A9972_23310	0	324	0	100	0	100
UMC65	A9978_04500	0	324	0	100	0	100
UME65	A9971_23290	0	324	0	100	0	100
SE9	A9970_06140	7.00E-31	350	49	27	208	94
VC13	A9977_15445	3.00E-169	322	0	71	92	99
**Strain**	**Best subject hit (locus_tag)**	**E-value**	**Alignment Length**	**no. gaps**	**ID (%)**	**no. mismatch**	**Coverage (%)**
**MRY70_06115: Putative multifunctional fatty acid oxidation complex subunit alpha**							
M1	MRY70_06115	0	707	0	100	0	100
CE77	A9975_16425	2.00E-94	663	35	32	414	93
UMA601	FCJ53_20045	0	707	0	100	0	100
UMA603	FCJ55_04745	0	707	0	100	0	100
UMA643	FCJ56_04420	0	707	0	100	0	100
UMC631	FCN13_00485	0	707	0	100	0	100
UMC3103	FCN10_11595	0	707	0	100	0	100
UMC3106	FCJ51_00485	0	707	0	100	0	100
UMC3129	FCN12_00485	0	707	0	100	0	100
UMC76	A9979_07010	0	707	0	100	0	100
UME83	A9972_23310	0	707	0	100	0	100
UMC65	A9978_04495	0	707	0	100	0	100
UME65	A9971_23285	0	707	0	100	0	100
SE9	A9970_25620	2.00E-71	649	39	27	438	90
VC13	A9977_15660	0	717	12	52	332	99
**Strain**	**Best subject hit (locus_tag)**	**E-value**	**Alignment Length**	**no. gaps**	**ID (%)**	**no. mismatch**	**Coverage (%)**
**MRY70_06120: Putative short-chain dehydrogenase/reductase**							
M1	MRY70_06120	0	255	0	100	0	100
CE77	A9975_06425	4.00E-103	255	3	60	100	99
UMA601	FCJ53_20040	0	255	0	100	0	100
UMA603	FCJ55_04740	0	255	0	100	0	100

UMA643	FCJ56_04425	0	255	0	100	0	100
UMC631	FCN13_00490	0	255	0	100	0	100
UMC3103	FCN10_11600	0	255	0	100	0	100
UMC3106	FCJ51_00490	0	255	0	100	0	100
UMC3129	FCN12_00490	0	255	0	100	0	100
UMC76	A9979_07010	0	255	0	100	0	100
UME83	A9972_23310	0	255	0	100	0	100
UMC65	A9976_20790	0	255	0	100	0	100
UME65	A9978_04490	0	255	0	100	0	100
SE9	A9970_02615	4.00E-28	228	13	36	134	88
VC13	A9977_05860	6.00E-104	255	3	61	96	99
**Strain**	**Best subject hit (locus_tag)**	**E-value**	**Alignment Length**	**no. gaps**	**ID (%)**	**no. mismatch**	**Coverage (%)**
**MRY70_06125: Putative epoxide hydrolase**							
M1	MRY70_06125	0	379	0	100	0	100
CE77	A9975_09955	3.00E-03	98	10	31	58	26
UMA601	FCJ53_20035	0	379	0	100	0	100
UMA603	FCJ55_04735	0	379	0	100	0	100
UMA643	FCJ56_04430	0	379	0	100	0	100
UMC631	FCN13_00495	0	379	0	100	0	100
UMC3103	FCN10_11605	0	379	0	100	0	100
UMC3106	FCJ51_00495	0	379	0	100	0	100
UMC3129	FCN12_00495	0	379	0	100	0	100
UMC76	A9979_07010	0	379	0	100	0	100
UME83	A9972_23310	0	379	0	100	0	100
UMC65	A9978_04485	0	379	0	100	0	100
UME65	A9971_23275	0	379	0	100	0	100
SE9	A9970_03200	9.00E-131	390	15	46	197	99
VC13	A9977_09985	1.00E-125	392	21	50	177	99
**Strain**	**Best subject hit (locus_tag)**	**E-value**	**Alignment Length**	**no. gaps**	**ID (%)**	**no. mismatch**	**Coverage (%)**
**MRY70_06130: Hypothetical protein**							
M1	MRY70_06130	0	198	0	100	0	100
CE77	A9975_06380	3.00E-19	175	8	33	109	86
UMA601	FCJ53_20030	0	190	0	100	0	95
UMA603	FCJ55_04730	0	190	0	100	0	95
UMA643	FCJ56_04435	0	190	0	100	0	95
UMC631	FCN13_00500	0	190	0	100	0	95
UMC3103	FCN10_11610	0	190	0	100	0	95
UMC3106	FCJ51_00500	0	190	0	100	0	95
UMC3129	FCN12_00500	0	190	0	100	0	95

UMC76	A9979_07010	0	190	0	100	0	95
UME83	A9972_23310	0	190	0	100	0	95
UMC65	A9978_04480	0	190	0	100	0	95
UME65	A9971_23270	0	190	0	100	0	95
SE9	A9970_28170	3.00E-01	82	9	27	51	41
VC13	A9977_07665	2.00E-11	173	18	27	108	82

**Strain**	**Best subject hit (locus_tag)**	**E-value**	**Alignment Length**	**no. gaps**	**ID (%)**	**no. mismatch**	**Coverage (%)**
**MRY70_06135: Putative regulatory protein from LuxR family**							
M1	MRY70_06135	0	876	0	100	0	100
CE77	A9975_00030	1.00E-155	911	47	37	524	99
UMA601	FCJ53_20025	0	876	0	100	0	100
UMA603	FCJ55_04725	0	876	0	100	0	100
UMA643	FCJ56_04440	0	876	0	100	0	100
UMC631	FCN13_00505	0	876	0	100	0	100
UMC3103	FCN10_11615	0	876	0	100	0	100
UMC3106	FCJ51_00500	0	876	0	100	0	100
UMC3129	FCN12_00505	0	876	0	100	0	100
UMC76	A9979_07010	0	876	0	100	0	100
UME83	A9972_23310	0	876	0	100	0	100
UMC65	A9978_04475	0	876	0	100	0	100
UME65	A9971_23265	0	876	0	100	0	100
SE9	A9970_06200	1.00E-07	99	9	33	57	11
VC13	A9977_15430	8.00E-161	891	30	38	523	99

**Strain**	**Best subject hit (locus_tag)**	**E-value**	**Alignment Length**	**no. gaps**	**ID (%)**	**no. mismatch**	**Coverage (%)**
**MRY70_06140: Putatve rubredoxin**							
M1	MRY70_06140	0	52	0	100	0	100
CE77	A9975_30015	2.00E-19	52	0	60	21	98
UMA601	FCJ53_20020	0	52	0	100	0	100
UMA603	FCJ55_04720	0	52	0	100	0	100
UMA643	FCJ56_04445	0	52	0	100	0	100
UMC631	FCN13_00510	0	52	0	100	0	100
UMC3103	FCN10_11620	0	52	0	100	0	100
UMC3106	FCJ51_00510	0	52	0	100	0	100
UMC3129	FCN12_00510	0	52	0	100	0	100
UMC76	A9979_07010	0	52	0	100	0	100
UME83	A9972_23310	0	52	0	100	0	100
UMC65	A9978_04470	0	52	0	100	0	100
UME65	A9971_23260	0	52	0	100	0	100
SE9	A9970_06200	3	24	0	42	14	45

VC13	A9977_04325	2.00E-16	51	0	53	24	96
**Strain**	**Best subject hit (locus_tag)**	**E-value**	**Alignment Length**	**no. gaps**	**ID (%)**	**no. mismatch**	**Coverage (%)**
**MRY70_06145: Putative fatty acid desaturase**							
M1	MRY70_06145	0	297	0	100	0	100
CE77	A9975_11270	3.00E-01	69	3	26	48	23
UMA601	FCJ53_20015	0	297	0	100	0	100
UMA603	FCJ55_04715	0	297	0	100	0	100
UMA643	FCJ56_04450	0	297	0	100	0	100
UMC631	FCN13_00515	0	297	0	100	0	100
UMC3103	FCN10_11625	0	297	0	100	0	100
UMC3106	FCJ51_00515	0	297	0	100	0	100
UMC3129	FCN12_00515	0	297	0	100	0	100
UMC76	A9979_07010	0	297	0	100	0	100
UME83	A9972_23310	0	297	0	100	0	100
UMC65	A9978_04465	0	297	0	100	0	100
UME65	A9971_23255	0	297	0	100	0	100
SE9	A9970_07715	3.00E-02	239	66	24	115	63
VC13	A9977_04410	2.00E-01	120	14	27	74	38
**Strain**	**Best subject hit (locus_tag)**	**E-value**	**Alignment Length**	**no. gaps**	**ID (%)**	**no. mismatch**	**Coverage (%)**
**MRY70_06150: Putative regulatory protein from LuxR family**							
M1	MRY70_06150	0	872	0	100	0	100
CE77	A9975_34060	3.00E-49	929	102	27	576	98
UMA601	FCJ53_20010	0	872	0	100	0	100
UMA603	FCJ55_04710	0	872	0	100	0	100
UMA643	FCJ56_04455	0	872	0	100	0	100
UMC631	FCN13_00520	0	872	0	100	0	100
UMC3103	FCN10_11630	0	872	0	100	0	100
UMC3106	FCJ51_00520	0	872	0	100	0	100
UMC3129	FCN12_00520	0	872	0	100	0	100
UMC76	A9979_07010	0	872	0	100	0	100
UME83	A9972_23310	0	872	0	100	0	100
UMC65	A9978_04460	0	872	0	100	0	100
UME65	A9971_23250	0	872	0	100	0	100
SE9	A9970_06770	6.00E-08	61	0	41	36	7
VC13	A9977_15175	5.00E-61	918	68	27	600	99
**Strain**	**Best subject hit (locus_tag)**	**E-value**	**Alignment Length**	**no. gaps**	**ID (%)**	**no. mismatch**	**Coverage (%)**
**MRY70_06155: Putative ferredoxin reductase**							
M1	MRY70_06155	0	407	0	100	0	100

CE77	A9975_01115	4.00E-52	396	7	34	254	96
UMA601	FCJ53_20005	0	407	0	100	0	100
UMA603	FCJ55_04705	0	407	0	100	0	100
UMA643	FCJ56_04460	0	407	0	100	0	100
UMC631	FCN13_00525	0	407	0	100	0	100
UMC3103	FCN10_11635	0	407	0	100	0	100
UMC3106	FCJ51_00525	0	407	0	100	0	100
UMC3129	FCN12_00525	0	407	0	100	0	100
UMC76	A9979_07010	0	407	0	100	0	100
UME83	A9972_23310	0	407	0	100	0	100
UMC65	A9978_04455	0	407	0	100	0	100
UME65	A9971_23245	0	407	0	100	0	100
SE9	A9970_18230	8.00E-06	141	7	27	96	33
VC13	A9977_10830	2.00E-113	410	10	47	206	98
**Strain**	**Best subject hit (locus_tag)**	**E-value**	**Alignment Length**	**no. gaps**	**ID (%)**	**no. mismatch**	**Coverage (%)**
**MRY70_06160: Putative acyl-CoA ligase (MyrC)**							
M1	MRY70_06160	0	516	0	100	0	100
CE77	A9975_00035	2.00E-164	508	8	49	253	97
UMA601	FCJ53_20000	0	516	0	100	0	100
UMA603	FCJ55_04700	0	516	0	100	0	100
UMA643	FCJ56_04465	0	516	0	100	0	100
UMC631	FCN13_00530	0	516	0	100	0	100
UMC3103	FCN10_11640	0	516	0	100	0	100
UMC3106	FCJ51_00530	0	516	0	100	0	100
UMC3129	FCN12_00530	0	516	0	100	0	100
UMC76	A9979_07010	0	516	0	100	0	100
UME83	A9972_23310	0	516	0	100	0	100
UMC65	A9978_04450	0	516	0	100	0	100
UME65	A9971_23240	0	516	0	100	0	100
SE9	A9970_09400	9.00E-14	387	49	22	253	69
VC13	A9977_31725	4.00E-84	504	23	35	306	95
**Strain**	**Best subject hit (locus_tag)**	**E-value**	**Alignment Length**	**no. gaps**	**ID (%)**	**no. mismatch**	**Coverage (%)**
**MRY70_06165: Alcohol dehydrogenase (MyrB)**							
M1	MRY70_06165	0	367	0	100	0	100
CE77	A9975_09320	2.00E-48	374	16	31	243	99
UMA601	FCJ53_19995	0	367	0	100	0	100
UMA603	FCJ55_04695	0	367	0	100	0	100
UMA643	FCJ56_04470	0	367	0	100	0	100
UMC631	FCN13_00535	0	367	0	100	0	100

UMC3103	FCN10_11645	0	367	0	100	0	100
UMC3106	FCJ51_00535	0	367	0	100	0	100
UMC3129	FCN12_00535	0	367	0	100	0	100
UMC76	A9979_07010	0	367	0	100	0	100
UME83	A9972_23310	0	367	0	100	0	100
UMC65	A9978_04445	0	367	0	100	0	100
UME65	A9971_23235	0	367	0	100	0	100
SE9	A9970_23280	2.00E-12	340	37	25	219	89
VC13	A9977_01625	2.00E-44	373	15	31	244	99

**Strain**	**Best subject hit (locus_tag)**	**E-value**	**Alignment Length**	**no. gaps**	**ID (%)**	**no. mismatch**	**Coverage (%)**
**MRY70_06170: Putative aldehyde dehydrogenase (MyrA)**							
M1	MRY70_06170	0	458	0	100	0	100
CE77	A9975_08315	9.00E-115	464	8	39	275	99
UMA601	FCJ53_19990	0	458	0	100	1	100
UMA603	FCJ55_04690	0	458	0	100	1	100
UMA643	FCJ56_04475	0	458	0	100	1	100
UMC631	FCN13_00540	0	458	0	100	1	100
UMC3103	FCN10_11650	0	458	0	100	1	100
UMC3106	FCJ51_00540	0	458	0	100	1	100
UMC3129	FCN12_00540	0	458	0	100	1	100
UMC76	A9979_07010	0	458	0	100	1	100
UME83	A9972_23310	0	458	0	100	1	100
UMC65	A9978_04440	0	458	0	100	1	100
UME65	A9971_23230	0	458	0	100	1	100
SE9	A9970_15200	1.00E-148	451	5	47	235	98
VC13	A9977_15955	9.00E-48	420	11	29	287	91

**Strain**	**Best subject hit (locus_tag)**	**E-value**	**Alignment Length**	**no. gaps**	**ID (%)**	**no. mismatch**	**Coverage (%)**
**MRY70_06175: Putative enoyl-CoA hydratase (MyrD)**							
M1	MRY70_06175	0	266	0	100	0	100
CE77	A9975_17790	6.00E-47	266	12	39	150	98
UMA601	FCJ53_19985	0	262	0	100	0	98
UMA603	FCJ55_04685	0	262	0	100	0	98
UMA643	FCJ56_04480	0	262	0	100	0	98
UMC631	FCN13_00545	0	262	0	100	0	98
UMC3103	FCN10_11655	0	262	0	100	0	98
UMC3106	FCJ51_00545	0	262	0	100	0	98
UMC3129	FCN12_00545	0	262	0	100	0	98
UMC76	A9979_07010	0	262	0	100	0	98
UME83	A9972_23310	0	262	0	100	0	98

UMC65	A9978_04435	0	262	0	100	0	98
UME65	A9971_23225	0	262	0	100	0	98
SE9	A9970_11820	2.00E-23	250	17	28	164	91
VC13	A9977_25370	3.00E-27	241	1	32	163	90

Strain	Best subject hit (locus_tag)	E-value	Alignment Length	no. gaps	ID (%)	no. mismatch	Coverage (%)
**MRY70_06180: Putative regulatory protein from LysR family**							
M1	MRY70_06180	0	309	0	100	0	100
CE77	A9975_36690	2.00E-34	294	12	33	184	93
UMA601	FCJ53_19980	0	309	0	100	0	100
UMA603	FCJ55_04680	0	309	0	100	0	100
UMA643	FCJ56_04485	0	309	0	100	0	100
UMC631	FCN13_00550	0	309	0	100	0	100
UMC3103	FCN10_11660	0	309	0	100	0	100
UMC3106	FCJ51_00550	0	309	0	100	0	100
UMC3129	FCN12_00550	0	309	0	100	0	100
UMC76	A9979_07010	0	309	0	100	0	100
UME83	A9972_23310	0	309	0	100	0	100
UMC65	A9978_04430	0	309	0	100	0	100
UME65	A9971_23220	0	309	0	100	0	100
SE9	A9970_13275	2.00E-34	302	23	27	198	97
VC13	A9977_24665	2.00E-147	292	1	68	91	94

Strain	Best subject hit (locus_tag)	E-value	Alignment Length	no. gaps	ID (%)	no. mismatch	Coverage (%)
**MRY70_06185: Putative lipoprotein**							
M1	MRY70_06185	0	83	0	100	0	100
CE77	A9975_28740	3.00E-01	37	0	35	24	44
UMA601	FCJ53_19975	1.00E-57	83	0	100	0	100
UMA603	FCJ55_04675	1.00E-57	83	0	100	0	100
UMA643	FCJ56_04490	1.00E-57	83	0	100	0	100
UMC631	FCN13_00555	1.00E-57	83	0	100	0	100
UMC3103	FCN10_11665	1.00E-57	83	0	100	0	100
UMC3106	FCJ51_00555	1.00E-57	83	0	100	0	100
UMC3129	FCN12_00555	1.00E-57	83	0	100	0	100
UMC76	A9979_07010	1.00E-57	83	0	100	0	100
UME83	A9972_23310	1.00E-57	83	0	100	0	100
UMC65	A9978_04425	1.00E-57	83	0	100	0	100
UME65	A9971_23215	1.00E-57	83	0	100	0	100
SE9	A9970_19390	3.00E-02	39	0	36	25	46
VC13	A9977_24705	3.00E-07	60	6	47	26	70

Table S6. Protein homology of genes located within the 76-kb integrative element, flanking the 28-kb genomic island, from strain M1. ID, Identity between query protein sequence and subject blast hit. Cov, percentage of the query sequence covered by the alignment. BLASTP was performed with the ICEberg (Integrative and Conjugative Elements) and NCBI databases.

Locus tag	Protein length	Blastp hit	E-value	no. gaps	ID %	Cov %	Accession	Source
MRY70_05920	418	Tyrosine-based site-specific recombinase [*Cupriavidus metallidurans* CH34] [&]	1.20E-218	0	78	100	CAD31406.1	ICEberg
		Tyrosine-type recombinase/integrase [*Pseudomonas aeruginosa*]	0	0	99	100	MBH9406755.1	NCBI
		Pmen_3484 [*Pseudomonas mendocina* ymp]	0	0	92	99	WP_012019490.1	*
		Aave_3366 [*Acidovorax avenae* subsp. *citrulli* AAC00-1]	0	0	85	94	ABM33924.1	*
MRY70_05925	91	Conserved hypothetical protein [*Cupriavidus metallidurans* CH34] [&]	3.20E-57	0	89	100	ABF08349.1	ICEberg
		Hypothetical protein [*Pseudomonas aeruginosa*]	8.00E-61	0	98	98	WP_065984898.1	NCBI
MRY70_05930	174	Conserved hypothetical protein [*Cupriavidus metallidurans* CH34] [&]	9.40E-96	0	87	100	ABF08350.1	ICEberg
		DNA-binding protein [*Pseudomonas aeruginosa*]	2.00E-117	0	97	99	WP_043545610.1	NCBI
MRY70_05935	128	Transcriptional regulator [*Bordetella petrii*]	1.00E-09	0	37	46	CAP41623.1	ICEberg
		AlpA family transcriptional regulator [*Pseudomonas stutzeri*]	2.00E-84	0	98	99	WP_044316124.1	NCBI
MRY70_06190	667	Conjugal transfer protein TraG [*Acidovorax* sp. KKS102] [$]	0	9	84	99	BAJ72254.1	ICEberg
		Conjugal transfer protein [*Cupriavidus oxalaticus*] [#]	0	1	87	99	CAD61149.1	ICEberg
		Conjugal transfer protein TraG [*Pseudomonas aeruginosa*]	0	0	96	100	WP_126547500.1	NCBI
MRY70_06195	154	Hypothetical protein [*Acidovorax* sp. KKS102] [$]	1.00E-90	1	84	99	BAJ72255.1	ICEberg
		Hypothetical protein [*Cupriavidus oxalaticus*] [#]	7.00E-88	0	89	88	CAD61150.1	ICEberg
		CopG family transcriptional regulator [*Pseudomonas aeruginosa*]	1.00E-99	0	99	95	WP_043545501.1	NCBI
MRY70_06200	317	Mating pair formation protein [*Cupriavidus oxalaticus*] [#]	0	4	82	99	CAD61151.1	ICEberg
		Conjugal transfer protein TrbB [*Acidovorax* sp. KKS102] [$]	0	0	87	93	BAJ72256.1	ICEberg
		P-type conjugative transfer TrbB [*Pseudomonas balearica*]	0	0	97	99	WP_061338604.1	NCBI
MRY70_06205	128	Mating pair formation protein[#]	1.00E-36	3	83	95	CAD61152.1	ICEberg
		Conjugal transfer protein TrbC [*Acidovorax* sp. KKS102] [$]	6.00E-36	0	88	95	BAJ72257.1	ICEberg
		Conjugal transfer protein TrbC [*Pseudomonas balearica*]	1.00E-78	0	97	99	WP_061338603.1	NCBI
MRY70_06210	90	Conjugal transfer protein TrbD [*Acidovorax* sp. KKS102] [$]	3.00E-50	0	89	90	BAJ72258.1	ICEberg
		VirB3 family type IV secretion system protein [*Pseudomonas chloritidismutans*]	1.00E-55	0	99	98	WP_023446477.1	NCBI
MRY70_06215	812	Mating pair formation protein [*Cupriavidus oxalaticus*] [#]	0	2	86	99	CAD61153.1	ICEberg
		Conjugal transfer protein TrbE [*Acidovorax* sp. KKS102] [$]	0	0	88	99	BAJ72259.1	ICEberg

		Conjugal transfer protein TrbE [*Pseudomonas aeruginosa*]	0	0	96	99	WP_043545491.1	NCBI
MRY70_06220	246	Mating pair formation protein [*Cupriavidus oxalaticus*] #	8.00E-118	8	69	96	CAD61154.1	ICEberg
		Conjugal transfer protein TrbJ [*Acidovorax* sp. KKS102] $	1.00E-114	0	77	89	BAJ72260.1	ICEberg
		P-type conjugative transfer protein TrbJ [*Pseudomonas aeruginosa*]	3.00E-108	0	92	100	WP_033992421.1	NCBI
MRY70_06225	110	Lipoprotein [*Acidovorax* sp. KKS102] $	7.00E-17	0	67	58	BAJ72261.1	ICEberg
		Hypothetical protein [*Cupriavidus oxalaticus*] #	7.00E-14	3	38	85	CAD61155.1	ICEberg
		Putative secreted protein	1.00E-55	0	81	99	CDH69579.1	NCBI
MRY70_06230	458	Mating pair formation protein [*Cupriavidus oxalaticus*] #	0	22	78	99	CAD61156.1	ICEberg
		P-type conjugative transfer protein TrbL [*Pseudomonas aeruginosa*]	0	0	96	95	WP_151163727.1	NCBI
MRY70_06235	235	Conjugal transfer protein TrbF [*Acidovorax* sp. KKS102] $	1.00E-153	0	86	99	BAJ72263.1	ICEberg
		Mating pair formation protein [*Cupriavidus oxalaticus*] #	7.00E-153	0	86	99	CAD61157.1	ICEberg
		Conjugal transfer protein TrbF [*Pseudomonas aeruginosa*]	2.00E-166	0	98	99	WP_043545485.1	NCBI
MRY70_06240	329	Mating pair formation protein [*Cupriavidus oxalaticus*] #	0	1	85	99	CAD61158.1	ICEberg
		Conjugal transfer protein TrbG [*Acidovorax* sp. KKS102] $	0	1	88	99	BAJ72264.1	ICEberg
		P-type conjugative transfer protein TrbG [*Pseudomonas stutzeri*]	0	0	97	99	WP_181084039.1	NCBI
MRY70_06245	425	Conjugal transfer protein TrbI [*Acidovorax* sp. KKS102] $	0	16	80	99	BAJ72265.1	ICEberg
		Mating pair formation protein [*Cupriavidus oxalaticus*] #	0	14	76	99	CAD61159.1	ICEberg
		TrbI/VirB10 family protein [*Pseudomonas stutzeri*]	0	0	99	92	WP_181084041.1	NCBI
MRY70_06250	82	Hypothetical protein [*Acidovorax* sp. KKS102] $	4.00E-42	0	88	84	BAJ72266.1	ICEberg
		Hypothetical protein [*Cupriavidus oxalaticus*] #	3.00E-40	0	79	88	CAD61160.1	ICEberg
		DUF2274 domain-containing protein [*Pseudomonas aeruginosa*]	1.00E-49	0	96	98	WP_043545479.1	NCBI

& ICEberg|388; CMGI-3 element from *Cupriavidus metallidurans* CH34; functional role unknown, with genes apparently involved in pentose metabolism.

* Song L, Pan Y, Chen S, Zhang X. 2012. Structural characteristics of genomic islands associated with GMP synthases as integration hotspot among sequenced microbial genomes. Comput Biol Chem. 36:62-70

# ICEberg|166; Tn4371 family, Tn4371 element from *Cupriavidus oxalatica* A5; involved in the degradation of biphenyl and 4-chlorobiphenyl.

$ ICEberg|46; Tn4371 family, ICE-KKS element from *Acidovorax* sp. KKS102; functional role unknown.

Supplementary Figures

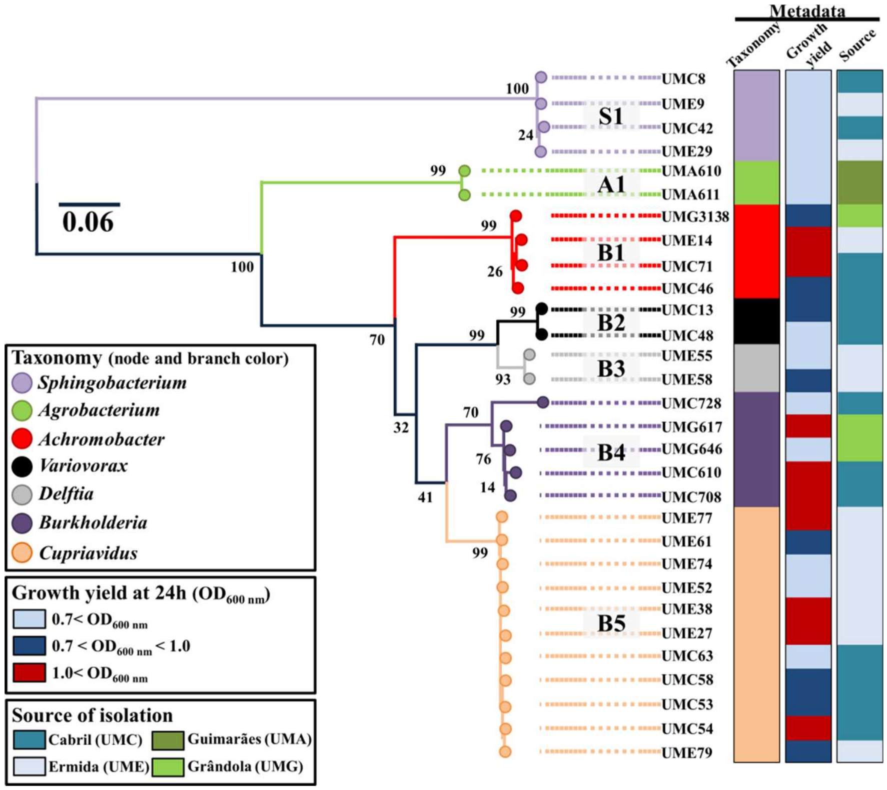

Fig. S1. Phylogeny of the bacterial strains belonging to *Sphingobacteriia*, *Alphaproteobacteria* and *Betaproteobacteria* classes, isolated from enrichment cultures of the rhizosphere soil samples from 4 geographic locations in Portugal. The phylogenetic midpoint rooted tree resulted from the multiple alignment of DNA sequences obtained by Sanger sequencing, comprising the V2 to V4 region of the 16S rRNA gene. The sequence of strain M1 was used as reference. Colored columns summarize the metadata associated with each isolate: tree taxonomy, source of isolation and the $OD_{600\ nm}$ yield registered after 24 h growth in 10 mL batch cultures of MM supplemented with β-myrcene as carbon source (Supplementary Table S4). Bootstrap values presented as percentage are shown in the tree branches. Isolate codes: "UMC", Cabril; "UME", Ermida; "UMA", Guimarães; "UMG", Grândola.

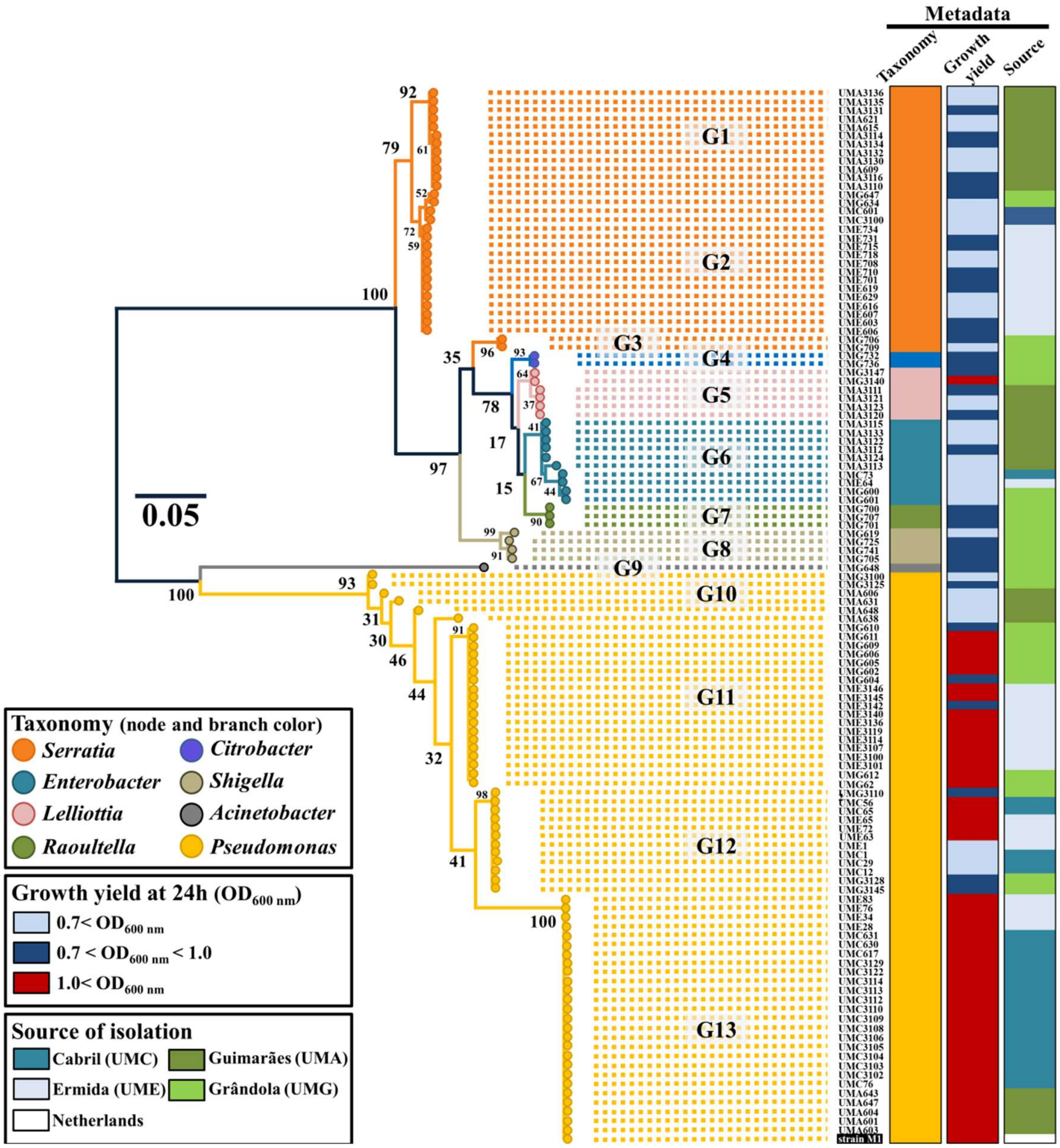

Fig. S2. Phylogeny of the bacterial strains belonging to *Gammaproteobacteria* class, isolated from enrichment cultures of the rhizosphere soil samples from 4 geographic locations in Portugal. The phylogenetic midpoint rooted tree resulted from the multiple alignment of DNA sequences obtained by Sanger sequencing, comprising the V2 to V4 region of the 16S rRNA gene. The sequence of strain M1 was used as reference. Colored columns summarize the metadata associated with each isolate: tree taxonomy, source of isolation and the $OD_{600\ nm}$ yield registered after 24 h growth in 10 mL batch cultures of MM supplemented with β-myrcene as carbon source (Supplementary Table S4). Bootstrap values presented as percentage are shown in the tree branches. Isolate's codes: "UMC", Cabril; "UME", Ermida; "UMA", Guimarães; "UMG", Grândola.
